# Unveiling the Adsorptive Potential of Natural Biopolymers for Olive Mill Wastewater Treatment: A Synergistic Approach Using RSM-BBD, Mixture Design, Kinetics, and Mechanistic Analysis

**DOI:** 10.3390/ijms26167738

**Published:** 2025-08-11

**Authors:** Sabah Elamraoui, Nouhaila Asdiou, Rachid El kaim Billah, Mounir El Achaby, Said Kounbach, Rachid Benhida, Mounia Achak

**Affiliations:** 1 Science Engineer Laboratory for Energy, National School of Applied Sciences, Chouaïb Doukkali University, El Jadida 24000, Morocco; elamraoui.s@ucd.ac.ma (S.E.); asdiou.n@ucd.ac.ma (N.A.); elkaimbillah.r@ucd.ac.ma (R.E.k.B.); 2 Materials Science, Energy, and Nano-Engineering (MSN) Department, Mohammed VI Polytechnic University (UM6P), Lot 660—Hay Moulay Rachid, Benguerir 43150, Morocco; mounir.elachaby@um6p.ma; 3 Chemical & Biochemical Sciences Green Process Engineering, CBS, Mohammed VI Polytechnic University, Ben Guerir 43150, Morocco; said.kounbach@um6p.ma (S.K.); rachid.b@um6p.ma (R.B.); 4 Institut de Chimie de Nice CRNS UMR7272, Université Côte d’Azur, 28 Avenue Valrose, 06108 Nice, France

**Keywords:** olive mill wastewater (OMW), lignocellulose, keratin, chitin, COD, adsorption

## Abstract

This study evaluates the structural properties and adsorption capacities of four bio-based adsorbents, sawdust (SD), straw (ST), chicken feathers (CFs), and shrimp shells (SSs), for chemical oxygen demand (COD) removal from olive mill wastewater (OMW). Response Surface Methodology (RSM) with a Box–Behnken Design (BBD) was applied to optimize the operational parameters, resulting in maximum COD uptake capacities of 450 mg/g (SD), 575 mg/g (ST), 700 mg/g (CFs), and 750 mg/g (SSs). Among these materials, SSs exhibited the highest COD removal efficiency of 85% under optimal conditions (pH 8, 20 g/L, 30 °C, 5 h, 111 rpm). A mixture design approach was then used to explore the synergistic effects of combining lignocellulosic (SD and ST), chitin-based (SSs), and keratin-based (CFs) adsorbents. The optimized blend (SD 10%, ST 28.9%, SS 38.3%, and CF 22.6%) achieved a COD removal efficiency of 82%, demonstrating the advantage of using mixed biopolymer systems over individual adsorbents. Adsorption mechanisms were investigated through isotherm models (Langmuir, Freundlich, Temkin, and Redlich–Peterson) and kinetic models (pseudo-first-order, pseudo-second-order, Elovich, and intraparticle diffusion). Lignocellulosic adsorbents predominantly followed physisorption mechanisms, while chitin- and keratin-rich materials exhibited a combination of physisorption and chemisorption. Thermodynamic analysis confirmed the spontaneous nature of the adsorption process, with SSs showing the most favorable Gibbs free energy (ΔG = −21.29 kJ/mol). A proposed mechanism for the adsorption of organic compounds onto the bio-adsorbents involves hydrogen bonding, electrostatic interactions, π–π interactions, n–π stacking interactions, hydrophobic interactions, and van der Waals forces. These findings highlight the potential of biopolymer-based adsorbents and their optimized combinations as cost-effective and sustainable solutions for OMW treatment.

## 1. Introduction

The circular economy has emerged as a key strategy in industrial production, aiming to minimize waste generation, promote resource efficiency, and reduce environmental impacts. This approach is particularly crucial in the agro-industrial sector, where large volumes of organic waste are generated [1,2]. Despite ongoing efforts to enhance waste valorization, certain industrial discharges remain highly polluting and difficult to manage. Among them, OMW is a serious environmental issue due to its high organic load, complex composition, and toxicity [3]. In recent decades, global olive oil production has expanded considerably, exceeding three million tons in 2018–2019, according to the International Olive Oil Council (IOOC) [4]. The organic load in OMW is primarily due to its exceptionally high chemical oxygen demand (COD) and biological oxygen demand (BOD_5_), which can reach 200 g/L and 60 g/L, respectively, far exceeding typical municipal wastewater levels [5]. It contains a variety of organic pollutants, including fatty acids, sugars, tannins, lignin, proteins, and polyphenols [3]. Polyphenols, which are present at concentrations of 0.5–20 g/L, contribute significantly to OMW toxicity due to their antimicrobial properties, which inhibit biological degradation processes [6]. As a result, its uncontrolled disposal leads to severe contamination of soil, groundwater, and aquatic ecosystems. In response to these challenges, various treatment strategies have been developed, including biological, physical, and chemical processes [7,8]. However, although methods such as coagulation, membrane filtration, and advanced oxidation processes have shown effectiveness, they are often associated with significant limitations, including high energy consumption, the use of costly reagents, and the generation of secondary waste [9,10,11]. Given these constraints, adsorption has emerged as a highly promising treatment method due to its simplicity, cost-effectiveness, and environmental sustainability [12]. Unlike other techniques, adsorption requires minimal energy input, operates under mild conditions, and can achieve high removal efficiencies. The effectiveness of adsorption largely depends on the nature and properties of the adsorbent, including its surface area, functional groups, and affinity for pollutants. Over the years, several types of adsorbents have been explored for OMW treatment, ranging from natural materials such as clays, banana peel, and spent coffee to synthetic commercial products like activated carbon and resins [13,14]. In addition, activation techniques for biomass were explored to enhance the adsorption efficiency by increasing the surface area and modifying the surface chemistry [15,16]. Furthermore, some studies suggest that the organic load can be selectively removed from OMW without external additives, demonstrating the potential of bio-based adsorbents in wastewater treatment [17,18,19]. However, direct efficiency comparisons between studies are challenging due to variations in OMW composition, initial COD and polyphenol concentrations, and experimental conditions. Numerous studies have extensively focused more on polyphenol adsorption from OMW on one adsorbent, but relatively few have focused on the broader removal of COD, which represents the total organic load [7,20]. To address these gaps and to compare the treatment performance of various bio-based adsorbents, this study systematically evaluates the adsorption behavior of four raw materials derived from lignocellulosic materials (sawdust (SD) and straw (ST)), chitin (shrimp shells (SSs)), and keratin-based adsorbents (chicken feathers (CFs)) in their raw states for the removal of COD from OMW. This comparative study, involving biopolymers derived from different sources, has not been previously reported. It highlights the potential of a wide range of materials as adsorbents and demonstrates how their distinct physicochemical properties and functional groups enable diverse interactions with various organic molecules. The complexity of COD, which comprises organic molecules of different polarity, size, and functionality, requires adsorbents with equally diverse surface chemistries. In addition to adsorption mechanisms, this study evaluates both the adsorption efficiency and large-scale availability of bio-based adsorbents. Lignocellulosic residues (sawdust, corn cobs, palm peats, sugarcane bagasse, argan shells, wheat straw, and rice straw), shrimp shells, and chicken feathers are produced in millions of tons annually and represent abundant, sustainable, and cost-effective resources for OMW treatment [21,22,23]. However, their combined use to enhance performance has been largely unexplored. A key innovation of this research is the exploration of synergistic effects through adsorbent combinations. This approach, which is widely applied in industrial process design, introduces a novel perspective on adsorption synergy and its potential to improve treatment efficiency [24]. To optimize the conditions for each individual material, Response Surface Methodology (RSM) with a Box–Behnken Design (BBD) was applied, allowing the systematic evaluation of the influence of operational parameters. Mixture experiments (MEs) were then conducted to assess the adsorption efficiency of various adsorbent blends and identify optimal compositions for maximum COD removal. Following optimization, adsorption isotherm, kinetic, and thermodynamic models were used to elucidate the underlying mechanisms. By combining a comparative analysis of individual biopolymers with a study of their synergistic effects, this research provides valuable insights into the adsorption potential of bio-based materials. The outcomes contribute to the development of cost-effective and high-performance treatment strategies, laying the groundwork for greener processes and supporting the principles of a circular economy.

## 2. Results and Discussions

### 2.1. OMW Characterization

The characterization of OMW in Table 1 revealed a substantial organic load, highlighting the need for effective treatment before disposal. The OMW used in this study had an acidic pH of 5.2, in line with values commonly cited in the literature. According to Dermeche et al. [25] and Azbar et al. [26], OMW typically has a pH between 2.24 and 5.9, primarily due to the presence of organic acids, phenolic compounds, and fermentation by-products [25,26]. Hence, the total chemical oxygen demand (COD) was measured at 223 g/L, with a dissolved COD fraction of 152 g/L and a biological oxygen demand (BOD_5_) of 28.50 g/L, indicating significant concentrations of biodegradable and non-biodegradable organic compounds. These values are higher than those reported by El-Gohary et al. [27], who found COD levels ranging from 45 to 150 g/L, suggesting variability due to differences in olive variety, extraction techniques, and seasonal factors. The polyphenol content, a key contributor to the toxicity of OMW, was found to be 3.11 g/L. This aligns with the findings of El Herradi et al. [28] but remains lower than the 5–10 g/L range according to Mekki et al. [29]. The presence of polyphenols inhibits microbial degradation, making biological treatment challenging and emphasizing the need for adsorption-based approaches [30]. In addition to its organic complexity, OMW also presents a significant inorganic fraction. The electrical conductivity (EC) of 15.79 mS/cm aligns with Aly et al. [31] but remains lower than the 20–43 mS/cm range noted by Niaounakis et Halvadakis [32]. The elevated conductivity is primarily attributed to the presence of salts, particularly NaCl, which is often added during the olive milling process to enhance preservation. Chloride (0.00215 g/L), phosphate (2.33 g/L), and potassium (0.00329 g/L) concentrations were consistent with the values observed by Venieri et al., 2010 [33]. However, the calcium (0.055 g/L) and magnesium (0.056 g/L) levels were lower than those reported by Akkam et al. [34], where the values often exceeded 100 mg/L, reflecting potential variability in soil composition and irrigation practices. Then, the sulfate concentration (2.72 g/L) was greater than the findings by Malvis et al. [35], emphasizing the potential contribution of OMW discharge to eutrophication in aquatic ecosystems. The total Kjeldahl nitrogen (TKN) concentration of 1.15 g/L, along with nitrite (0.00083 g/L) and nitrate (0.01218 g/L), indicate a substantial nitrogen load, which can contribute to algal blooms and oxygen depletion if discharged untreated [36]. Regarding the solid fraction, the total solids (TSs) were measured at 55.45 g/L, with total suspended solids (TSSs) at 3.25 g/L and fixed solids (FSs) at 33.18 g/L. These values are slightly lower than those provided by Vaz et al. [37], where TSs often exceeded 60 g/L, likely due to sedimentation differences during storage conditions. The high solid content contributes to turbidity and potential sediment accumulation, further complicating OMW management.

### 2.2. Characterization of the Adsorbents

#### 2.2.1. Proximate Analysis

The proximate analysis in Table 2 shows that SSs have a higher moisture content of 6%, suggesting a greater capacity for swelling and structural flexibility, followed by SD (4.5%), ST (3.24%), and CFs (3%), indicating good initial dryness and the potential for a high adsorption capacity. The ash content is significantly higher in CFs (4.5%) and SSs (3%) than in ST (2.96%) and SD (0.5%). This high ash content could suggest the presence of mineral components like calcium carbonate or phosphates, which might influence the adsorption properties by reducing the available active sites. Volatile matter is slightly higher in CFs (76.5%) than in others, while the fixed carbon content is higher in ST (23.57%), indicating a greater proportion of carbon-based structures. The component analysis highlights that ST has a much higher lignin content (39.45%) compared to sawdust (20.32%), making it more rigid and hydrophobic. The cellulose content is significantly greater in SD (60.47%) than in straw (35.79%), providing more hydroxyl (-OH) groups, which could enhance adsorption. The hemicellulose content is slightly higher in ST (20.47%) than in SD (15.63%), affecting porosity and water retention. The extractives are relatively low in both materials, with ST containing 4.29% and SD 3.58%, indicating minimal interference from non-structural compounds.

#### 2.2.2. Boehm Titration

The surface functional groups of ST, SD, CFs, and SSs were determined using the Boehm titration method, and the results are presented in Table 3. A notable difference in surface chemistry between the lignocellulosic materials (ST and SD) and the keratin-chitin based materials (CFs and SSs) was observed. For example, ST, with its high lignin content, has the largest number of acid groups (7.38 mmol/g), consisting mainly of carboxylic (4.75 mmol/g) and phenolic (1.98 mmol/g) acid groups. This high acidity suggests a strong potential for adsorbing cationic species and interacting with polar organic compounds via hydrogen bonding and electrostatic interactions, while its relatively low basic group content indicates that its surface is predominantly acidic. Likewise, SD contains mild acid groups (5.42 mmol/g), with carboxylic (3.25 mmol/g) and phenolic (1.12 mmol/g) acid groups. In comparison, keratin-based materials such as CFs have lower acid group concentrations than SSs, with 3.55 and 5.61 mmol/g for CFs and SSs, respectively, but both include significant lactone groups. On the other hand, keratin–chitin materials show a higher concentration of basic groups, with 2.12 and 3.17 mmol/g for CFs and SSs, respectively. This basic nature likely stems from the presence of amine and thiol groups, which may enhance affinity for acidic pollutants or facilitate adsorption through electrostatic attraction in acidic media [38,39]. In contrast, lignocellulosic materials such as ST and SD contain fewer basic groups, with 0.75 and 1.25 mmol/g for ST and SD, respectively. Consequently, the results indicate that lignocellulosic materials contain more acid groups, making them more suitable for adsorbing cations, while keratin–chitin materials, which are more basic, are more suitable for adsorbing anions.

#### 2.2.3. Isoelectric pH

**Figure 1:**
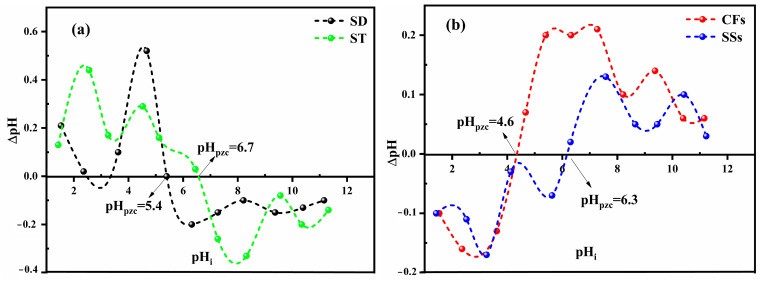
pH_pzc_ of the SD, ST (**a**), CF, and SS (**b**) adsorbents (SE = ±1.11% as error bars).

The isoelectric point (pH_pzc_) of all material represents the pH where the surface charge is neutral, which has an influence on its adsorption behavior. The pH_pzc_ values shown in Figure 1 demonstrate that the lignocellulosic materials (SD and ST) and keratin–chitin materials (CFs and SSs) display different surface charge properties. Notably, in Figure 1a, SD (pH_pzc_ = 5.4) becomes negatively charged at pH values above 5.4, while ST (pH_pzc_ = 6.7) carries positive charges below pH 6.7. This indicates that ST retains a positive surface charge over a wider pH range under acidic conditions compared to SD. Alternatively, Figure 1b shows that CFs exhibit a neutral surface charge at pH_pzc_ = 4.6, indicating that they become negatively charged at lower pH values compared to the other adsorbents. This behavior is consistent with their lower content of acidic functional groups, as confirmed by Boehm titration. Conversely, SSs, with a pH_pzc_ of 6.3, perform similarly to ST, keeping a positive charge over a wider pH range. In general, SD and CFs have lower pH_pzc_ values, which makes them more susceptible to acquire negative charges under slightly acidic conditions, while ST and SSs retain positive charges over a broader acidic pH range.

#### 2.2.4. FTIR and XRD

**Figure 2:**
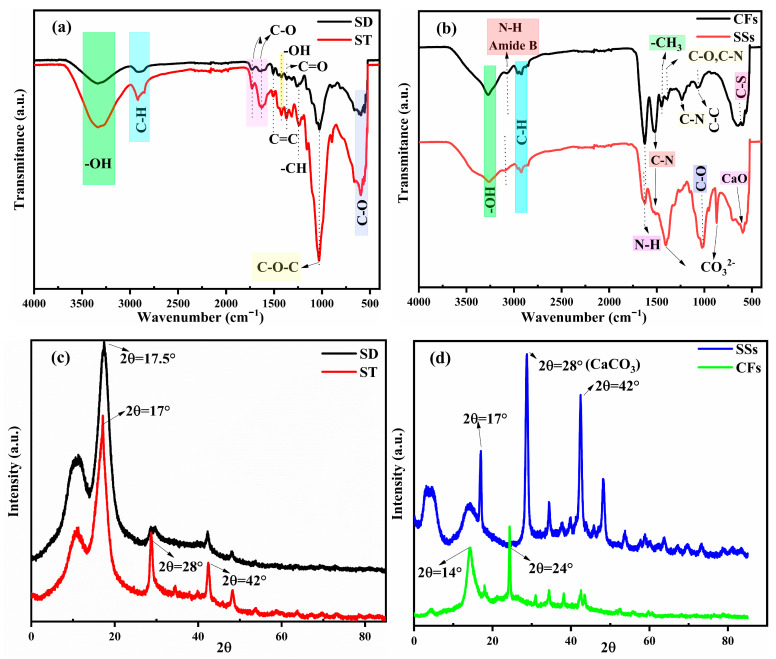
FTIR and XRD spectra for SD, ST (**a**,**c**), CF, and SS (**b**,**d**).

The FTIR spectra of sawdust (SD) and straw (ST) in Figure 2a display characteristic functional groups associated with lignocellulosic materials, especially cellulose, hemicellulose, and lignin. Both materials show a broad O-H stretching band around 3327 cm^−1^, pointing to the occurrence of hydroxyl groups in cellulose and hemicellulose. C-H stretching vibrations occur at 2888 cm^−1^ (SD) and 2913 cm^−1^ (ST), suggesting slight variations in aliphatic structures [40]. In the carbonyl region, both materials show a C=O stretching band at 1727 cm^−1^, assigned to ester and carboxyl functional groups, principally hemicellulose and lignin [41,42]. However, ST reveals additional peaks at 1627 cm^−1^ and 1619 cm^−1^, linked to aldehydes, lactones, ketones, and carboxyl groups, indicating a higher degree of oxidation or structural differences in the lignin content. Similarly, aromatic backbone vibrations (C=C) at 1505 cm^−1^ appear in both SD and ST, confirming the presence of lignin [43]. Polysaccharide-related C-O-C stretching vibrations are observed at 1257 cm^−1^ (SD) and 1237 cm^−1^ (ST), with C-O alkoxy groups at 1024 cm^−1^ (SD) and 1031 cm^−1^ (ST) [44]. This shift reflects differences in the cellulose and hemicellulose composition. In addition, out-of-plane aromatic C-H bending at 893–862 cm^−1^ (SD) and 899 cm^−1^ (ST) confirms the presence of lignin. Overall, despite the fact that both materials present common lignocellulosic characteristics, ST has stronger carbonyl and oxidation-related peaks, indicating a higher degree of structural modification, while SD appears to have slightly more marked lignin-associated vibrations. The CFs and SSs contain other structures and compositions than SD and ST due to their dominant biopolymers, keratin and chitin, respectively. The spectrum in Figure 2b displays broad O-H and N-H stretching bands around 3263 and 3267 cm^−1^, respectively, which are characteristic of proteins and polysaccharides, as reported by Nandiyanto et al. [45], and associated with aromatic amide II vibrations. The C-H stretching vibrations (2959 and 2924 cm^−1^) appear in both spectra, reflecting the presence of CH_2_ and CH_3_, respectively, though the variations in intensity suggest slight differences in the hydrocarbon content. A major distinction is observed in the N-H stretching region at 1628 cm^−1^, where the peak intensity is notably higher in the SS sample compared to CFs. This stronger absorption in SSs is attributed to the presence of amide I vibrations typical of chitin, whereas in CFs, the corresponding band is mainly associated with secondary amide bonds present in keratin [46]. The amide II band (1511–1532 cm^−1^ in SSs vs. 1523 cm^−1^ in CFs) also exhibits slight shifts, reflecting differences in the hydrogen bonding and secondary structures of chitin and keratin. Additionally, SSs exhibit an absorption band at 1403 cm^−1^, which could be associated with carbonate ions or more likely attributed to carboxylate (-COO^−^) groups, as noted by Gbenebor et al., confirming the presence of CO_3_^2−^ in crustacean-derived chitin [47]. Meanwhile, CFs show peaks at 1447 and 1392 cm^−1^, which may correspond to CH_3_ groups and the C-N bond of amide III [48]. Significant differences are also observed in the C-O stretching region, SSs exhibit strong peaks at 1019 and 951 cm^−1^, indicative of C-O bonds in polysaccharides, reinforcing its chitin composition, whereas CFs display peaks at 1232 and 1063 cm^−1^, which may be linked to the peptide bonds C-N and C-C, respectively [49]. Furthermore, SSs contain distinct low-frequency bands at 866 cm^−1^, 697 cm^−1^, and 594 cm^−1^, which are absent in CFs. These peaks correspond to carbonate (CO_3_^2−^) and calcium-based compounds (CaO), confirming the mineral content of SSs, a distinguishing factor from CFs [47]. Meanwhile, CFs contain a band at 618 cm^−1^ indicative C-S vibration due to the presence of cysteine–disulfide bonds [50,51].

The XRD analysis of SD and ST reveals notable differences in their structural characteristics (Figure 2c). SD exhibits a broad hump around 2θ = 20–25°, indicative of its predominantly amorphous lignocellulosic structure, where cellulose, hemicellulose, and lignin contribute to the disordered arrangement. In contrast, ST displays sharper peaks superimposed on the amorphous background, suggesting the presence of some crystalline phases, potentially due to silica or mineral impurities naturally occurring in ST. Comparing SSs and CFs (Figure 2d), SSs exhibit sharp and well-defined peaks, particularly around 2θ = 26°, 29°, 39°, and 48°, confirming the presence of crystalline calcium carbonate (CaCO_3_) in calcite and aragonite forms, typical of biogenic mineralized structures. On the other hand, CFs show broad, less intense peaks, characteristic of keratin-based materials, with a dominant amorphous arrangement near 2θ = 20° reflecting the disordered proteinaceous structure.

#### 2.2.5. BET

As shown in Figure S1, the N_2_ adsorption–desorption isotherms for the investigated materials exhibit significant differences due to their structural diversity. Among the samples, only SSs displays a well-defined isotherm, classified as Type II, characteristic of non-porous or macroporous solids with unrestricted monolayer–multilayer adsorption. This is consistent with its relatively high adsorption capacity and the progressive filling of surface sites at higher relative pressures. The other materials (SD, ST, and CFs) do not present well-defined isotherm shapes. CFs most closely resemble a Type III isotherm, which is indicative of weak adsorbate–adsorbent interactions and low surface affinity for nitrogen. ST and SD exhibit irregular adsorption–desorption curves with no distinct inflection points, reflecting limited porosity and poorly developed textural properties. Because the desorption branch lies below the adsorption branch for all samples, an interpretation of hysteresis loops is not appropriate and has been omitted. Overall, these results highlight the superior textural characteristics of SSs compared to the other adsorbents, in agreement with its higher surface area and adsorption performance. In terms of surface area (Table 4), ST (5.3215 m^2^/g) has the highest value, making it the most efficient among the studied materials, followed by SSs (1.6740 m^2^/g), which benefits from its mesoporous structure. CFs (0.5648 m^2^/g) and SD (0.2810 m^2^/g) exhibit the lowest values, limiting their N_2_ adsorption potential. These results are in line with earlier studies on raw lignocellulosic materials, whereby the comparatively low BET surface area is attributed to the compact structure and limited accessibility of internal pores preceding chemical modification [52]. The BJH pore size distribution in Figure S2 further supports this trend, with CFs (6.757 nm) and SSs (5.895 nm) having larger mesopores, which facilitate the adsorption of bigger molecules, whereas ST (3.822 nm) and SD (3.688 nm) have smaller mesopores, contributing to their varying adsorption behaviors. Additionally, ST has the highest pore volume (0.0813 cm^3^/g), followed by SD (0.0583 cm^3^/g), SSs (0.0455 cm^3^/g), and CFs (0.0305 cm^3^/g), indicating that ST provides the most accessible porous network. This comparison highlights the superior adsorption properties of ST among lignocellulosic materials and SSs among protein-based adsorbents, with SD being the least effective due to its compact nature and limited porosity.

#### 2.2.6. SEM and EDX

**Figure 3:**
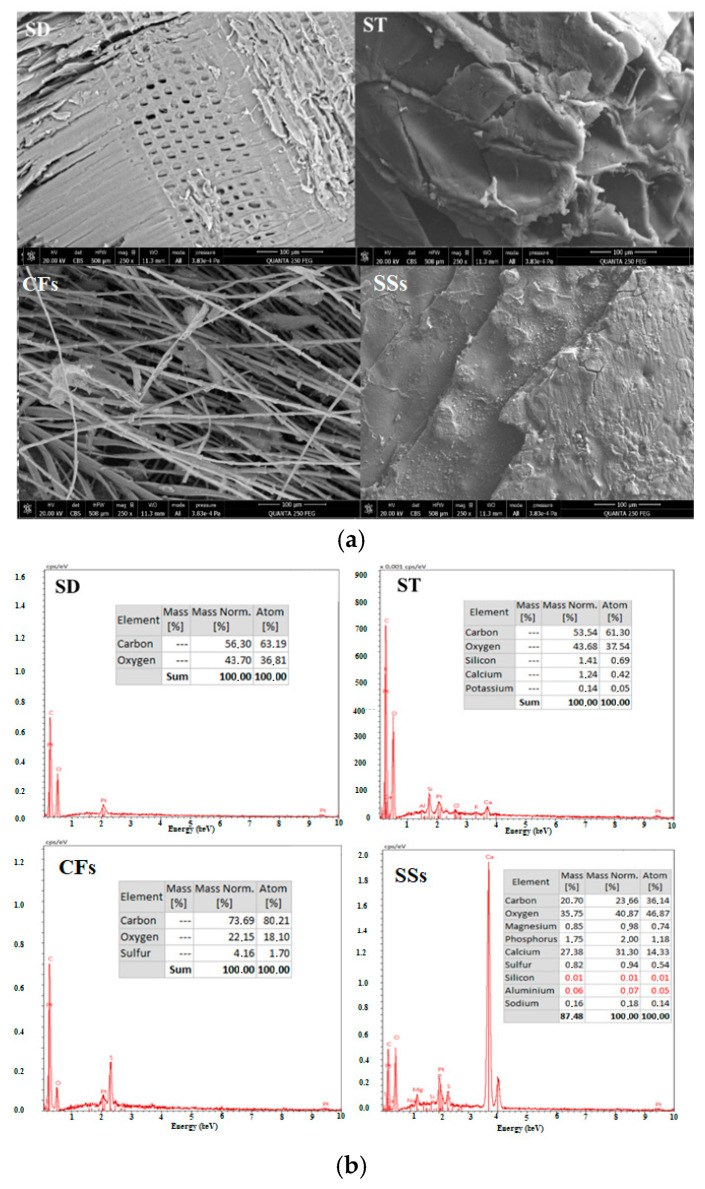
(**a**) SEM micrographs of the samples SD, ST, CFs, and SSs; (**b**) EDX of the samples SD, ST, CFs, and SSs.

The SEM analysis in Figure 3a reveals distinct morphological features influencing the adsorption properties of each material. ST exhibits an open cavity structure, which improves surface accessibility and explains its higher adsorption performance compared to SD, despite SD displaying vessel-like porous structures. CFs consist of an interwoven fibrous network, creating slit-shaped mesopores that contribute to adsorption but may limit overall capacity. SSs feature a lamellar surface with visible cracks, indicative of their chitinous composition, which enhances mesoporosity and capillary condensation. An identical morphological appearance of SSs was explained by Gbenebor et al. [47] as a sheet-like fibril texture. Therefore, EDX analysis provides an informative insight into the elemental composition of the adsorbents in Figure 3b, complementing the morphological observations of SEM. As lignocellulosic materials, SD and ST are primarily composed of carbon (56.30% and 53.54%, respectively) and oxygen (43.70% and 43.68%). Notably, ST also contains trace amounts of inorganic elements such as silicon, calcium, and potassium, likely derived from soil residues or the plant’s inherent mineral content [53]. In contrast, CFs, consisting mainly of keratin, have the highest carbon content (73.69%) and the lowest oxygen content (22.15%), as well as sulfur (4.16%), which is characteristic of keratin due to the presence of cysteine–disulfide bonds [50]. Hence, SSs rich in chitin and calcium carbonate have a much lower carbon content (20.70%), while oxygen (35.75%) and calcium (27.38%) dominate, reflecting the presence of calcium minerals [54]. The presence of magnesium, phosphorus, and sulfur in SSs confirms their biomineralized structure. These characteristics variations play a crucial role in defining the adsorption potential of each material.

### 2.3. COD Removal

#### 2.3.1. BBD Experimental Results

Adsorption experiments were conducted under varying conditions to evaluate the efficiency of different adsorbents (ST, SD, CFs, and SSs) in removing COD from OMW (Table S4). The experimental results showed that the COD removal yield varied significantly across different trials, with values ranging from 9.25% to 88.54% for ST, 13.70% to 69.40% for SD, 11.28% to 84.99% for CFs, and 10.52% to 85.36% for SSs. These variations reflect the influence of different operational parameters, including pH (A), adsorbent mass (B), temperature (C), contact time (D), and stirring speed (E). The wide variation in COD removal efficiency highlights the influence of the experimental conditions on the adsorption performance. A quadratic regression analysis was performed to fit response functions to the experimental data. Moreover, quadratic effects (A^2^, B^2^, C^2^, D^2^, and E^2^) were considered to assess the non-linear influences of these parameters on COD removal. The diagnostic plots of normal probability plots and externally studentized residuals in Figure S3 confirmed the adequacy of the fitted models for all adsorbents (SD, ST, CFs, and SSs). In the normal probability plots (Figure S3a), the residuals were distributed approximately along a straight line, indicating that the assumption of normality was satisfied. Moreover, the externally studentized residuals shown in Figure S3b were randomly scattered within the acceptable limits (±3), showing no apparent trends or outliers. According to the ANOVA results in Table S5, the non-linear model was highly significant for all adsorbents (*p* < 0.0001). pH (A) exhibited the most pronounced effect on COD removal across all adsorbents, with F-values of 295.08, 312.67, 233.10, and 85.13 for ST, SD, CFs, and SSs, respectively, confirming its dominant role in the adsorption process. For ST, temperature (C) was a significant factor (F = 84.79, *p* < 0.0001), along with the adsorbent mass (B) (F = 11.43, *p* = 0.0024). In contrast, contact time (D) and stirring speed (E) had negligible effects. For SD, only pH (A) showed a strong impact, while the other factors were statistically insignificant. The results for CFs demonstrated the influence of pH (A) and temperature (C) (F = 8.39, *p* = 0.0077), while SSs were significantly influenced by stirring speed (E) (F = 14.99, *p* = 0.0007), indicating that higher agitation improved the removal efficiency. Interaction effects were also notable, particularly AC for ST (F = 14.20, *p* = 0.0009), BE for SSs (F = 9.10, *p* = 0.0058), and DE for SSs (F = 46.59, *p* < 0.0001), signifying complex dependencies between parameters. The quadratic term of pH (A^2^) was significant for all adsorbents, confirming its non-linear effect on COD removal. Overall, the study highlights the crucial role of pH in the adsorption process, while other parameters such as temperature, stirring speed, and mass exhibited adsorbent-dependent effects.

Table S6 presents several key statistical metrics that assess the quality and reliability of the quadratic model for COD removal (Equations (1)–(4)) fit for all adsorbent tested (ST, SD, CFs, SSs) by the parameters as follows: R^2^, R^2^_Adj_, R^2^_Pred_, CV, AICC, and BIC. The ST and SD models perform best overall, with ST having the highest R^2^ (0.950) and R^2^_Adj_ (0.911), suggesting it fits the data well and predicts with good accuracy. SD, though having a slightly lower R^2^ (0.941), has the best AICC and BIC scores, making it the most efficient model. Then, CFs with the highest CV and lowest R^2^_Pred_, are the least consistent and predictive, indicating that they might not be the best choice for reliable modeling. Moreover, SSs have some drawbacks with higher AICC (395.49) and BIC values (395.39), implying that they might be too complex without offering much improvement in fit or prediction.

COD(SD) = 28.93 − 19.71A − 6.43AB + 11.32A^2^ + 3.84D^2^ (1)

COD(ST) = 51.87 − 25.65A + 13.87C − 11.25AC + 6.34AD + 7.60BC + 7.88BE − 14.65A^2^ − 4.34C^2^ − 5.05E^2^ (2)

COD(CFs) = 29.35 + 28.16A − 5.34C − 6.52BD + 7.09DE + 17.06A^2^ + 7.34D^2^ + 5.38E^2^ (3)

COD (SSs) = 59.16 + 13.80A + 7.56E − 12.88AC − 22.26DE − 29.70A^2^ − 7.73D^2^ (4)

#### 2.3.2. Lignocellulose Materials: Sawdust and Straw

**Figure 4:**
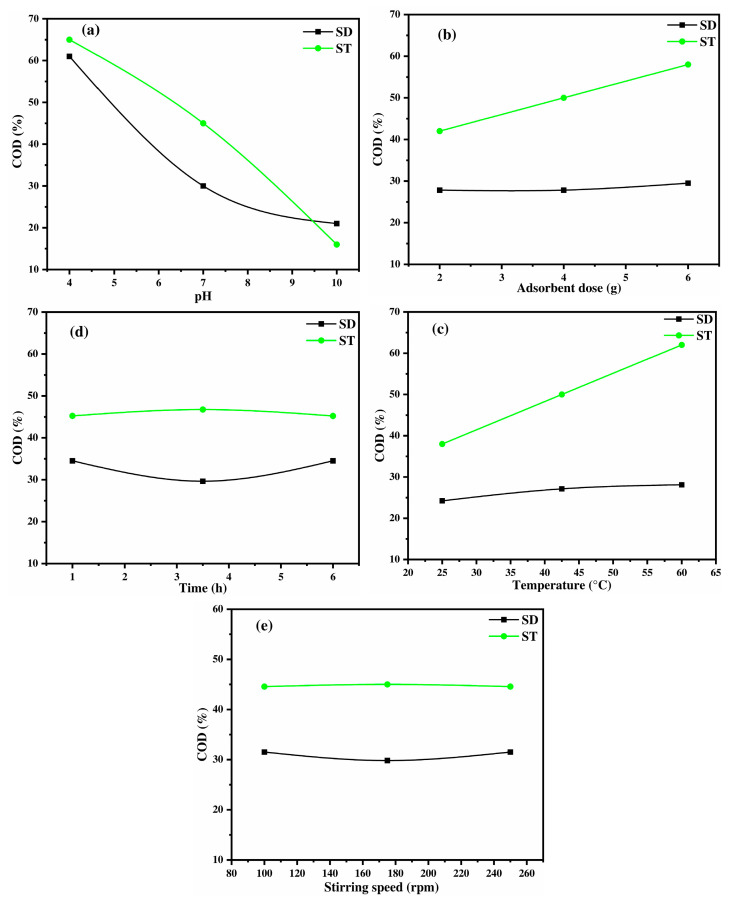
Evaluation of COD adsorption by ST and SD under varying conditions (**a**) pH, (**b**) mass, (**c**) time, (**d**) temperature and (**e**) stirring speed effect using BBD (SE = ±2.25% as error bars).

The efficiency of COD removal by SD and ST is largely governed by their common biopolymer components: cellulose, hemicellulose, and lignin. These constituents provide essential functional groups, particularly hydroxyl (-OH), carboxyl (-COOH), and phenolic (-PhOH) groups, which drive the adsorption process. Nevertheless, differences in the structural arrangement and concentration of these functional groups can cause variations in adsorption performance. It is essential to investigate the impact of these compositional variations on the efficiency of COD removal. The variation in pH, an essential parameter for comparing the two adsorbents, is illustrated in Figure 4a. The results show that the COD removal efficiency decreases as the pH increases for both materials. However, ST consistently exhibits higher removal efficiency than SD. At acidic pH (4–6), both adsorbents perform effectively, with SD achieving 60% and ST reaching 65%, likely due to the protonation of functional groups, which enhances their interactions with organic compounds. In contrast, at alkaline pH (8–10), the adsorption efficiency declines sharply, dropping to 20% for SD and 16% for ST. This decrease is primarily attributed to electrostatic repulsion between the negatively charged adsorbent surfaces and organic molecules, which hinders adsorption. The higher efficiency of ST compared to SD can be attributed to its greater availability of active sites, as confirmed by the Boehm titration results showing a higher content of acidic functional groups in ST (Table 3). These acidic groups improve its competitiveness in adsorbing negatively charged organic adsorbates from OMW. The strong dependence of adsorption on pH further underscores the importance of electrostatic interactions; under acidic conditions (pH 4–5), the protonation of functional groups enhances their interaction with contaminants such as phenolate ions (C_6_H_5_O^−^) as a part of the organic load from OMW, leading to improved COD removal. This behavior is consistent with previous studies conducted by Al Bsoul et al. [55], who report enhanced adsorption of organic pollutants at lower pH values using titanium oxide nanoparticles. Meanwhile, according to Figure 1, the isoelectric points of SD (pH_pzc_ = 5.4) and ST (pH_pzc_ = 6.7) suggest that their surfaces become positively charged under acidic conditions, enhancing the adsorption of negatively charged adsorbates. On the opposite, at higher pH levels, an increased organic OMW content, solubility, and competition with hydroxyl ions progressively reduce the adsorption efficiency, resulting in lower COD removal [12]. The adsorbent dose effect (Figure 4b) confirms that increasing the available surface area and functional groups improves adsorption, particularly for ST, which exhibits a more pronounced response. An increase from 40 to 65% is observed by adding 2 to 6 g of ST adsorbent, while SD remains nearly constant due to the limited reactive sites on this material compared with ST. In line with previous observations by Bargaoui et al. [56], the increase in COD removal by the rising adsorbent mass of ST can be attributed both to the increased number of surface retention sites and to the interstitial pores between particles that operate as effective scavengers for organic matter. These finding are supported by morphological and physicochemical characterizations. The SEM analysis revealed an open cavity structure with interconnected pores, facilitating the diffusion and trapping of organic molecules. Subsequently, the BET surface indicated a non-uniform surface adsorption characterized by multilayer adsorption, promoting more adsorption layers without pore saturation. Moreover, the presence of lignin potentially contributes to π-π interactions with the aromatic components of organic matter. Thus, the synergistic effects of the surface area, pore structure, and chemical functionalities in ST explain the improvement in COD removal with increasing adsorbent mass.

Furthermore, the positive impact of temperature on ST shows an increase from 37 to 63% by increasing the temperature from 25 to 60 °C (Figure 4c). It suggests an endothermic adsorption process, where thermal energy enhances the interaction between pollutants and adsorption sites, while SD remains less temperature sensitive, likely due to structural arrangement differences. For instance, Elayadi et al. [57] reported enhanced adsorption of polyphenols onto sugarcane bagasse with increasing temperature, attributing this trend to the greater mobility of adsorbate molecules and the activation of additional surface binding sites. Similarly, Bargaoui et al. [56] observed that temperature positively influenced COD adsorption on cypress sawdust, particularly due to the improved diffusion of organic matter into micropores and the expansion of pore channels at higher temperatures. In contrast, the limited temperature sensitivity of SD aligns with results noted by Papaoikonomou et al. [58], who demonstrated that adsorbents with less developed porosity and fewer functional sites exhibit minimal improvements in adsorption performance at higher temperatures due to steric hindrance, exothermic characteristics, and a low internal diffusion capacity. Therefore, both adsorbents reach their maximum efficiency at around 3–4 h of 30% and 45% of COD removal for SD and ST, respectively (Figure 4d). ST shows a slight increase and stabilization at longer contact times, while SD exhibits a slight decline after 3 h, likely due to the saturation of available sites. The equilibrium treatment duration observed for ST and SD is consistent with the findings of Djeziri et al. [59] for activated carbon, underscoring the competitiveness of ST and SD as effective materials for COD removal. Similarly, Elayadi et al. [60] and Boumediene et al. [61] reported that equilibrium for polyphenol and COD adsorption using agricultural waste-based adsorbents was reached within 2 to 4 h, supporting the kinetic efficiency of both ST and SD. However, stirring speed has a negligible impact from 100 to 250 rpm, as shown in Figure 4e, reinforcing that the process is more influenced by chemical interactions than mass transfer limitations. This result is in accordance with Elayadi et al. [60], who reported that increasing the agitation speed from 80 to 300 rpm negatively affected organic load removal efficiency. They found that the optimal stirring speed for maximum uptake (49.6%) was 80 rpm, while the optimal stirring speed for maximum COD removal (20%) was 300 rpm. These results suggest that higher stirring speeds may disrupt the adsorption equilibrium, potentially due to desorption effects or the breakdown of adsorbent–adsorbate interactions. The superior performance of ST compared with SD can be ascribed to the greater accessibility of functional groups, probably due to variations in the lignin content. Our experimental data suggest that ST has a higher lignin content than SD, leading to greater availability of adsorption sites, as proved in Table 2. As demonstrated in Table 3, ST exhibited a greater abundance of acidic functional groups than SD, as confirmed by Boehm titration. Specifically, ST presented a total of 7.38 mmol/g of acid groups, comprising 4.75 mmol/g of carboxylic groups and 1.98 mmol/g of phenolic groups. This result aligns with previous research proving that the lignin composition significantly affects the adsorption capacity [62,63]. Ultimately, the common composition of both adsorbents enables efficient COD removal, but differences in structural properties and surface chemistry make ST a more effective material for OMW treatment.

#### 2.3.3. Keratin-Based Material: Chicken Feathers

**Figure 5:**
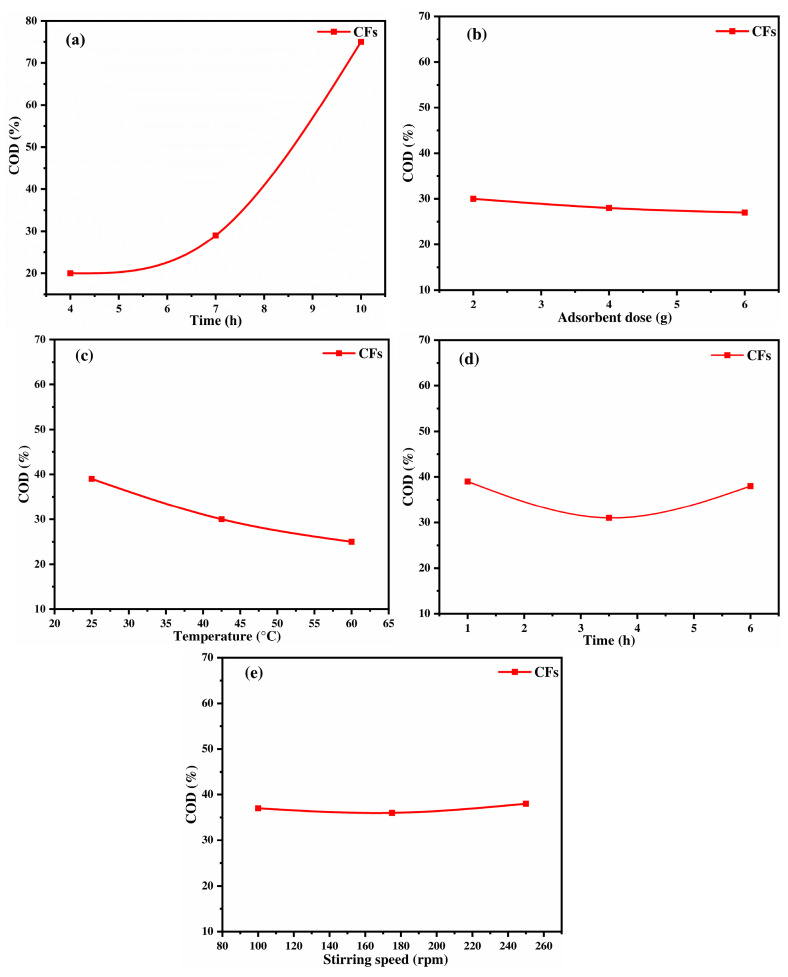
Evaluation of COD adsorption by CFs under varying conditions (**a**) pH, (**b**) mass, (**c**) time, (**d**) temperature and (**e**) stirring speed effect using BBD (SE = ±2.25% as error bars).

The COD removal efficiency using CFs is strongly influenced by the physicochemical properties of keratin, the primary component of this material [64]. Keratin functional groups, rich in heteroatoms such as nitrogen and sulfur, impact the adsorption process due to their electronegativity and ability to interact with pollutants [65,66]. The pH variation plays a dominant role, with COD removal rising from 20% at pH 4 to 75% at pH 10, reaching its highest point in basic medium (Figure 5a). This trend can be attributed to the deprotonation of carboxyl (-COOH) and amine (-NH_2_) groups in keratin, which results in a negatively charged surface, as proved by isoelectric point found at pH_pzc_ = 4.6 illustrated in Figure 1 [67,68]. Since OMW contains a mixture and complex compounds with different pK_a_ values, a combination of neutral and ionic forms coexists in solution, leading to multiple adsorption mechanisms. At high pH, organic pollutants, including fatty acids, polysaccharides, lignin, proteins, alcohols, aldehydes, and phenolic compounds, exist mainly in their anionic forms. These molecules have different proton-binding sites among them: carboxyl group (-COOH, pK_a1_ = 6.2), amide group primary amine (-NH_2_, as in amino acids, pK_a2_ = 9.5), secondary amine (-NH, pK_a2_ = 8.5), phenol group (pK_a3_ = 9.95) and hydroxyl group (-OH pK_a4_ ≥ 12). Under these alkaline conditions, enhanced deprotonation increases the overall negative charge of the organic molecules, thereby intensifying electrostatic repulsion with the negatively charged CF surface (pH_pzc_ = 4.6) and impacting the adsorption process [68]. This aligns with previous findings, where total phenol removal increased with pH, particularly when activated carbon was used as an adsorbent [69]. This finding is in alignment with the phenol adsorption study conducted by Banat and Al-Asheh [21], who utilized CFs at the optimal pH of 10. However, this finding is in disagreement with the study reported by Nassar et al. [12], who proposed the existence of positively charged sodium phenoxide ions capable of interacting with negatively charged surfaces through electrostatic interactions. In addition, other interactions, such as hydrogen bonding, van der Waals forces, and π-π interactions, may also contribute to the adsorption process. The adsorbent dose shows a stable trend of COD removal at 30% from 20 to 60 g/L in Figure 5b, indicating that surface saturation occurs beyond a certain mass, where additional CFs do not significantly improve adsorption. This suggests that the adsorption capacity is optimized at lower doses. As optimized by Banat and Al-Asheh [21] for phenol adsorption, no detectable phenol remained in the solution at the end of the sorption process up to 4 g/L. This trend can be related to the distinctive physicochemical characteristics of CFs; the FTIR spectrum confirmed the presence of keratin with the amide II band at 1523 cm^−1^, indicating protein-based structures capable of forming hydrogen bonds with organic molecules. EDX analysis also revealed a high carbon content (73.69%) and a notable sulfur content (4.16%), characteristic of keratin cysteine disulfide bridges, which enhance CF affinity for COD removal via van der Waals interactions and hydrogen bonding. In addition, SEM observations showed a fibrous, entangled surface morphology with slit-like mesopores, which facilitate initial adsorption. However, at higher doses, limited pore accessibility and potential particle aggregation can hinder further adsorption, explaining the plateau observed in COD removal. Consequently, temperature has a negative effect on COD removal (Figure 5c), indicating that the adsorption process is exothermic. When the temperature rises from 25 °C to 60 °C, it can weaken hydrogen bonds and reduce interactions between the pollutants and the adsorbent, resulting in a lowering of the adsorption efficiency from 40% to 25% [70]. Additionally, keratin’s rigid structure, rich in disulfide bonds, limits flexibility in functional groups, affecting pollutant interaction at elevated temperatures [66]. Therefore, contact time follows a typical kinetic trend, with an initial increase in COD removal as active sites interact with pollutants, followed by equilibrium at t = 1 h (Figure 5d). A similar equilibrium time has been found by Caovilla et al. [71], where the amount of adsorbed dye increases progressively with contact time before stabilizing. For instance, equilibrium was observed to be reached after around 90 min, with the active adsorption sites saturated. A slight decline at extended contact times may result from desorption or competitive displacement of weakly bound molecules. This behavior supports the findings of Hansen et al. [72], as they found that natural protein-based adsorbents such as chicken feathers exhibit rapid adsorption kinetics owing to the high reactivity of functional groups (amine, carboxyl, and sulfur) on keratin chains, which facilitate electrostatic interactions and hydrogen bonding with organic matter. However, saturation of these sites and possible conformational rearrangements of keratin fibrils over time can lead to a reduction in adsorption efficiency beyond equilibrium. Stirring speed has a negligible effect between 100 and 250 rpm, as shown in Figure 5e, reinforcing that the adsorption process is primarily governed by chemical interactions rather than external mass transfer limitations. This result is consistent with Elayadi et al. [19], who found that increasing the stirring speed had a negative impact on polyphenol removal, likely due to desorption effects. Overall, these findings highlight the adsorption potential of CFs, where keratin functional groups play an important role in pollutant binding.

#### 2.3.4. Chitin-Biobased Polymer: Shrimp Shells

**Figure 6:**
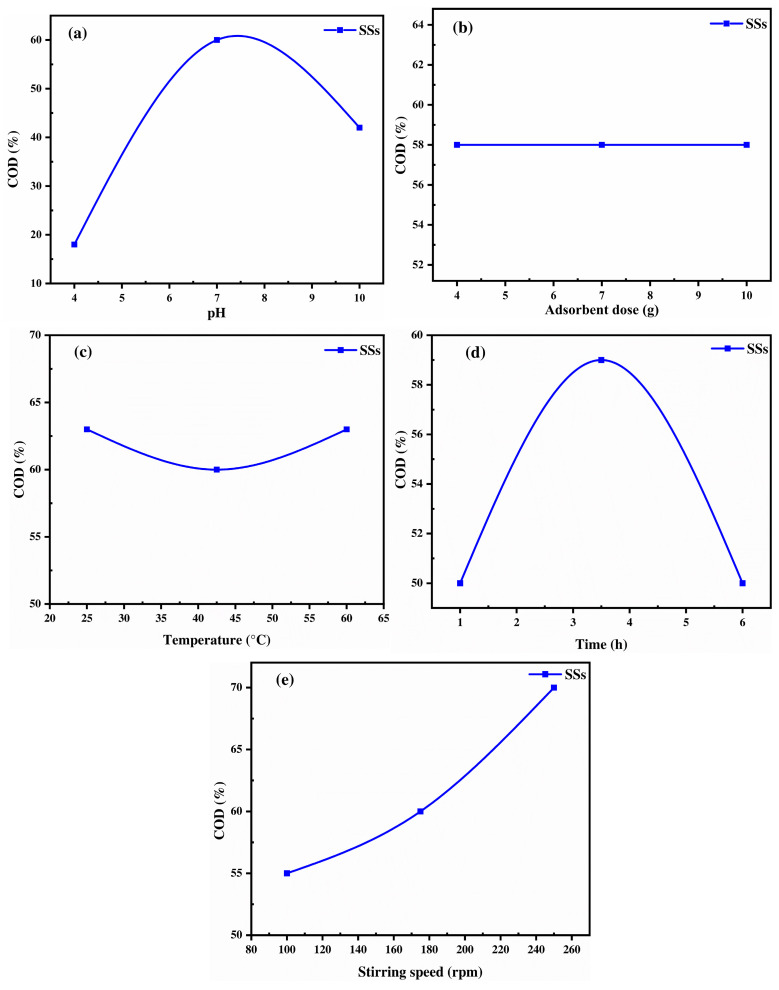
Evaluation of COD adsorption by SSs under varying conditions (**a**) pH, (**b**) mass, (**c**) time, (**d**) temperature and (**e**) stirring speed effect using BBD (SE = ±2.25% as error bars).

The adsorption behavior of SSs for COD removal from OMW is governed by their biopolymeric composition, primarily chitin [73]. Additionally, proteins and calcium carbonate (CaCO_3_) each contribute distinct functional groups to the adsorption process [74,75]. The pH strongly influences adsorption efficiency, with COD removal increasing from 20% at pH 4 to a peak at pH 7 = 8 (65%), before declining at higher pH (Figure 6a). This trend is attributed to the protonation and deprotonation of functional groups in SSs, particularly amine (-NH_2_) and hydroxyl (-OH) groups in chitin, as well as by considering its point of zero charge (pH_pzc_ = 6.3). At acidic pH (pH = 4–7), the surface of the SSs is positively charged due to the protonation of amine (-NH_2_) groups, forming =NH_3_^+^ and creating strong electrostatic attraction with negatively charged organic pollutants such as deprotonated carboxyl (-COO^−^) and phenolic (-O^−^) groups [76]. However, at pH > 7, the surface of the SSs becomes negatively charged due to the deprotonation of functional groups and increased repulsion between negatively charged pollutants. Therefore, proteins in shrimp shells contribute carboxyl (-COOH) and amide (-CONH_2_) as functional groups, which may also engage in hydrogen bonding, influencing adsorption performance, as determined in the FTIR analysis by a large functional group content. These findings align with the study by Geetha Devi et al. [76], who reported a COD removal efficiency of 67% under acidic conditions using chitosan. However, they contradict the finding of Elayadi et al. [77], who reported a higher COD removal efficiency (43%) under basic OMW conditions when using shrimp shells as an adsorbent. This discrepancy may be attributed to differences in experimental design. In our study, the Box–Behnken Design (BBD) matrix was employed, allowing for a more accurate determination of the optimal conditions through a quadratic model, which accounts for curvature effects by incorporating three levels of variation. In contrast, the linear factorial design used in the study by Elayadi et al. [77] may not have adequately captured the complex interactions influencing adsorption performance.

Concerning the adsorbent dose, Figure 6b shows that increasing the dose from 20 to 60 g/L does not significantly affect COD removal, which remains constant at around 57%. This suggests that beyond a certain concentration, the adsorption sites become saturated, and adding more adsorbent does not enhance the removal efficiency [78]. This could be due to particle aggregation, leading to reduced surface area availability or steric hindrance, preventing further pollutant adsorption. Meanwhile, temperature appears to have a slight negative effect on adsorption, supporting the exothermic nature of the process. As the temperature increases from 25 °C to 42.5 °C, a minor decrease in COD removal is observed from approximately 63% to 60%, after which it remains relatively stable up to 60 °C (Figure 6c) [79]. This reduction is likely due to disruption of hydrogen bonds between organic pollutants and chitosan hydroxyl (-OH) and amine (-NH_2_) groups, as well as the weakening of van der Waals interactions and possible desorption of previously adsorbed molecules at higher temperatures. The contact time has a significant impact on COD removal, with efficiency increasing from 49% at 1 h to 59% at 3 h, where equilibrium is reached (Figure 6d). Beyond this point, the efficiency decreases to 48% at 6 h, likely due to adsorption site saturation, limiting the further uptake of organic matter. Furthermore, repulsive forces between adsorbed pollutants and those still present in the bulk solution could hinder further adsorption [17]. This suggests that an optimal contact time of 3 h ensures maximum adsorption while preventing potential desorption effects. Moreover, the stirring speed enhances adsorption from 100 to 250 rpm, with COD removal improving from 55% to 72% (Figure 6e), likely due to increased mass transfer, better adsorbent dispersion, and greater exposure of active sites for pollutant binding [80]. To compare the efficiency of all materials for COD removal and the influence of operational conditions, especially pH, it is important to consider their surface chemistry. Chitin- and keratin-based adsorbents tend to perform better in alkaline environments because their amino groups lose protons and develop negative charges, which improves their attraction to positively charged or neutral pollutants. On the other hand, cellulose-based materials contain hydroxyl groups that remain protonated under acidic conditions, giving their surface a positive charge that enhances the binding of negatively charged substances.

### 2.4. COD Removal Optimization by RSM

The RSM optimization results highlight significant variations in adsorption efficiency among the four adsorbents, demonstrating their distinct adsorption behaviors for COD removal under different conditions. For COD removal in Figure S4**,** CFs (86.27%) and SSs (85.71%) achieved the highest efficiencies at pH 10 and 8, respectively, under moderate temperatures and prolonged contact times (5 h), emphasizing the role of pH in optimizing pollutant removal. Hence, ST demonstrated strong COD adsorption (82%) at pH 4, T = 55 °C, and t = 4 h, These findings underscore that CFs and SSs are the most efficient adsorbents, excelling in COD removal, while SD exhibits selective adsorption efficiencies, showing a balanced performance of 69.81%.

### 2.5. Synergetic Effect of the Adsorbents for COD Removal

#### 2.5.1. Statistical Analysis

The analysis of adsorption performance across 15 experimental runs, as detailed in Table S7, reveals a synergistic interaction between lignocellulosic adsorbents (SD (A) and ST (B)) and keratin- and chitin-based adsorbents (SSs (C) and CFs (D)), resulting in a significant improvement in COD removal efficiency. The highest COD removal (92.13%) is observed in combination five (SD = 0.1, ST = 0.4, CFs = 0.1, and SSs = 0.4), suggesting that the mixture of ST and CFs enhances organic matter adsorption. This can be attributed to the complementary interaction between the high surface area and mesoporosity of ST determined by the BET analysis and the functional protein groups of CFs, which contribute to the stronger adsorption of organic pollutants. Interestingly, the following mixture (SD = 0.25, ST = 0.25, CFs = 0.25, and SSs = 0.25), where all adsorbents are equally mixed, results in a moderate COD removal (54.56%), suggesting that while a balanced mixture provides a stable adsorption capacity, specific combinations of adsorbents exhibit stronger synergetic effects. Conversely, runs dominated by a single adsorbent such as Run 10 (SD = 0.7, 39.65% COD removal) and Run 14 (CFs = 0.7, 27.58% COD removal) show lower removal efficiencies, underscoring the limitations of using individual adsorbents alone. These findings align with those of Santi et al. [81], who also reported a synergistic effect on COD adsorption when using inorganic materials such as soil–bentonite and zeolite, emphasizing their high retention capacity in the treatment of OMW. Similarly, other studies have identified cotton and zeolite as an effective composite adsorbent for polyphenol removal, reinforcing the importance of material combinations in optimizing adsorption performance [31]. The modeling of the synergistic effect was conducted to describe COD adsorption, and full quadratic and cubic models were tested to evaluate response variations based on the experimentally established combinations.

The ANOVA results for COD uptake demonstrate distinct adsorption behaviors influenced by adsorbent interactions (Table S8). The quadratic model is statistically significant (*p* = 0.0003), with a strong linear mixing effect (*p* < 0.0001) indicating that overall adsorbent configuration plays a key role. Significant interactions, in especially AD (*p* = 0.0040), BC (*p* = 0.0003) and CD (*p* = 0.0180), point to a synergistic effect between SD-CFs, ST-SSs and SSs-CFs, respectively. In Table S9, the cubic model does not fit well due to the absence of significant higher-order interactions, implying that COD removal involves a simpler interactive mechanism, as determined by quadratic variation. Meanwhile, the strong fit was confirmed in Table S10 by the high correlation coefficient (R^2^ = 0.988, R^2^_adjusted_ = 0.966) for COD removal by the quadratic model and none by the cubic model due to low predictability (R^2^_Pred_ = −0.23). However, the predicted R^2^ of 0.8112 for COD removal indicate a moderate drop in predictive accuracy by the quadratic effect assigned to AD, BC, and CD.

#### 2.5.2. Contour Plot and 3D Response Surface Analysis of COD Removal by Mixture Design

**Figure 7:**
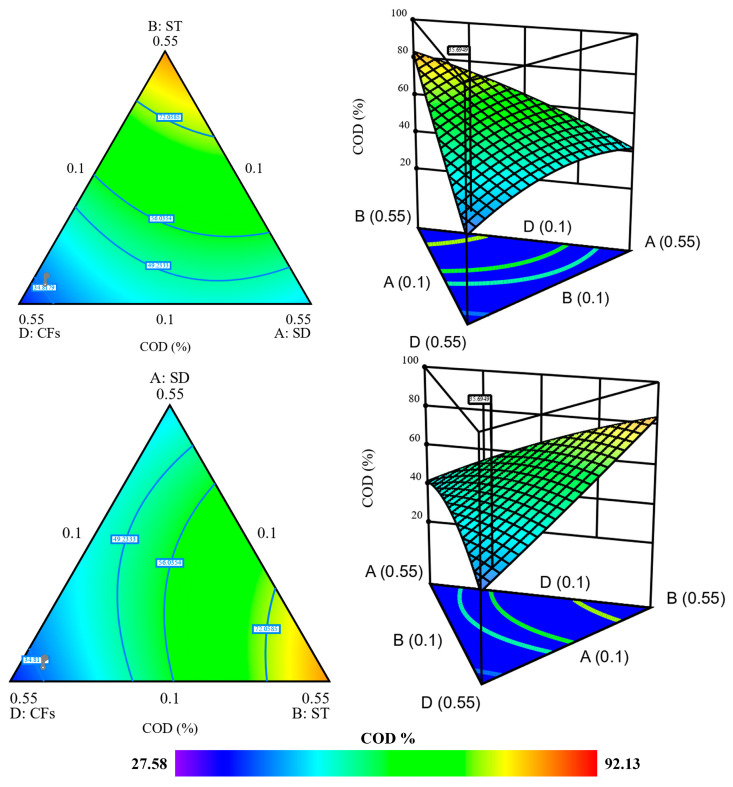
Contour plot and 2D and 3D response surface variations for COD removal by adsorbent mixtures (blue color: low, green: moderate, and orange-red color: high removal).

The contour plot and 3D response surface illustrate the synergistic interactions among the adsorbents for COD removal, revealing distinct adsorption behaviors based on the composition of the mixture. For COD removal in Figure 7, the highest efficiency of 82% and 80% is observed in the ABD mixture (55% ST, 10% SD, and 35% CFs) and ACD (55% SSs, 10% SD, and 35% CFs), respectively. This highlights the strong contributions of ST and SSs in adsorbing organic matter, and indicating that CFs alone do not provide sufficient adsorption capacity for COD. Intermediate removal efficiencies (40–50%) are recorded for mixtures like ABD (55% SD, 10% ST, and 35% CFs), suggesting that SD contributes moderately to adsorption but does not dominate the process. The mixture design approach effectively identified the optimal combination for high COD removal, in contrast to the study by Odeh et al. [82], who employed a mixture of magnetite, goethite, and dodecyl dimethyl ammonium without evaluating the interaction effects between the components.

#### 2.5.3. Optimization of the Mixture Design for the Synergetic Effect of the Adsorbents

In the provided graph in Figure S5, the deviations from the reference blend expressed in pseudo-units illustrate how changes in the proportion of each adsorbent (A (SD), B (ST), C (SSs), and D (CFs)) impact the efficiency of COD uptake and removal. The trends indicate that increasing or decreasing the amounts of individual adsorbents leads to variations in adsorption performance in the mixture involved. A (SD) and D (CFs) in the mixture exhibit a strong decreasing trend, while components B and C show a positive influence. The optimized adsorbent mixture for the COD response was determined to be A = 10%, B = 28.9%, C = 38.3%, and D = 22.6%, featuring a harmonious composition that maximizes both COD removal efficiencies at 82%, which has never been studied or reported for raw or activated material composites to find the optimal mixture for COD capture from OMW [83]. This optimal formulation likely takes advantage of the synergistic interactions between lignocellulosic and keratin–chitin combinations, which improve adsorption efficiency through a variety of surface functionalities.

### 2.6. Isotherm and Kinetics for COD Removal

According to the isotherm data presented in Table S11, each adsorbent displayed distinct adsorption behaviors toward COD removal from OMW. CFs demonstrated a remarkable performance, as confirmed by the highest Langmuir maximum capacity of Q_m_ = 839 mg/g and a strong model fit with R^2^ = 0.99, reflecting efficient monolayer adsorption on a homogeneous surface. The high K_L_ of 0.060 supports a significant affinity between CFs and COD, meanwhile the Freundlich constants (K_F_ = 184.7, n_F_ = 3.38) imply a heterogeneous surface with favorable multilayer adsorption. Likewise, SSs achieved a high Q_m_ of 821 mg/g with a consistent fit represented by the Langmuir model R^2^ (0.99) and K_L_ (0.064), indicating strong and uniform site interactions. The Freundlich values of K_F_ = 192.5 and n_F_ = 3.52 reinforce the favorable, heterogeneous nature of adsorption, while the Temkin constant of B = 163.01 reflects notable interactions between the adsorbate and adsorbent. The Redlich–Peterson model confirms the existence of a combined adsorption mechanism for CF and SS (R^2^ = 0.99). In contrast, ST showed a lower adsorption capacity of Q_m_ = 292.14 mg/g but a higher K_L_ of 1.98, suggesting high site-specific affinity but limited surface availability. Then, Freundlich parameters (K_F_ = 202, n_F_ = 1.89) and Temkin constant with a moderate binding energy of B = 72.2 indicate less efficient adsorption compared to CFs and SSs. On the other hand, SD showed the weakest adsorption performance with a Q_m_ of 386 mg/g and a very low K_L_ of 0.001, resulting in a R_L_ value above one, pointing to unfavorable adsorption. The low Freundlich constants of K_F_ =5.53 and n_F_ = 1.08, and high Temkin B value of 232.2 suggest minimal interactions and limited surface functionality. In summary, CFs and SSs proved to be the most effective adsorbents for COD removal, as displayed by their high adsorption capacities, strong affinities, and excellent fit with isothermal models. Their superior efficiency can be ascribed to the significant presence of nitrogen- and sulfur-containing functional groups, as revealed by the FTIR analysis, as well as to their dual acid–base surface character confirmed by Boehm titration. Likewise, ST and especially SD showed lower adsorption efficiencies, probably due to a lower number of active sites and weaker surface interactions. Based on the available literature, the Q_m_ value obtained in the present study is higher than that of other materials reported in previous research. Based on the available literature, the Q_m_ value obtained in the present study is higher than those reported for various other materials used for similar purposes. Specifically, Elayadi et al. [60] reported a Q_m_ value of 331.92 mg/g for sugarcane bagasse in the removal of COD from OMW. In another study, Achak et al. [17] used banana peel for the reduction of phenolic compounds from OMW, achieving a Q_m_ of 688.9 mg/g. Similarly, Xiaoli and Youcai [84] investigated the use of age–refuse for phenol removal from OMW and reported a Q_m_ value of 205.84 mg/g. These comparisons underscore the enhanced adsorption capacity of the material investigated in the present study. Based on the kinetic data presented in Table S12, the adsorption behavior of COD onto the four adsorbents was comprehensively analyzed using four kinetic models: pseudo-first-order (PFO), pseudo-second-order (PSO), Elovich, and intraparticle diffusion. For SD and ST, the PFO model provided the best fit, with R^2^ = 0.971 and 0.926, respectively, indicating that physisorption is likely to be dominant, although the PSO model also showed a good fit, with R^2^ = 0.93 and 0.90, respectively. In fact, the Elovich model showed strong correlations, with R^2^ = 0.970 and 0.87, indicating the presence of a heterogeneous surface of SD and ST, respectively, and pointing toward predominantly physical and surface-controlled adsorption. However, the intraparticle diffusion model had a low R^2^ of 0.69, suggesting a limited contribution of pore diffusion. In contrast, CFs displayed high compatibility with both the PFO (R^2^ = 0.974) and PSO (R^2^ = 0.970) models, with a particularly high theoretical adsorption capacity (q_e_ = 998 mg/g from PSO), suggesting a combined mechanism involving both physical and chemical adsorption. Almost analogous work has claimed that organic load adsorption is best described by the PSO model, as reviewed by Elayadi et al. [77] and Al Bsoul et al. [55]. Meanwhile, the intraparticle diffusion plot (R^2^ = 0.931) indicated a significant contribution from pore diffusion. Similarly, SSs showed excellent agreement with both the PFO (R^2^ = 0.983) and PSO (R^2^ = 0.978) models, along with high Elovich and intraparticle model fits (R^2^ = 0.968 and 0.934, respectively), suggesting a complex multi-step process with a significant chemisorption component. Comparatively, CFs and SSs demonstrated the most rapid and efficient adsorption kinetics, with high K_diff_ values (43.45 and 41.62 mg/g·min^1/2^), highlighting the importance of surface interaction in the initial phase.

### 2.7. Thermodynamic Study of COD and Polyphenols

The comparison was established to verify the feasibility, spontaneity, and nature of the adsorption process under different temperatures conditions for COD removal. The low negative Gibbs free energy (ΔG) for all materials was different at each temperature tested in the adsorption test. Table S13 indicates the spontaneous nature of adsorption, with SSs (−24.92 kJ/mol) presenting the highest spontaneity at 25 °C and SD (−9.826 kJ/mol) the lowest at 25 °C due to endothermic characteristic of this material, as confirmed by favorability at higher temperature. Moreover, the unspontaneity of SD at 25 °C is due to positive value of ΔG =16.232 kJ/mol). The enthalpy change (ΔH) varies between adsorbents, with SD and ST showing positive values of 70.843 kJ/mol and 30.030 kJ/mol, respectively, indicating endothermic adsorption that increases with increasing temperature. This could be ascribed to the increased mobility of the organic load, which allows them to acquire more kinetic energy and promotes their diffusion from the bulk solution to the solid phase as the temperature increases. Additionally, the higher temperature increases the number of active surface sites available for adsorption, that improving the adsorption process [85]. In contrast, CFs and SSs have negative ΔH values (−18.374 kJ/mol and −26.255 kJ/mol, respectively), suggesting exothermic adsorption, which may decrease at higher temperatures. Comparable results were reported by Elayadi et al. [57], suggesting an exothermic process for sugarcane bagasse as an adsorbent. Furthermore, the entropy change (ΔS) also varies, with ST showing positive values (144.66 J/mol·K), reflecting increased randomness at the solid–liquid interface, promoting adsorption. However, CFs, SD, and SSs display negative values, pointing to a more orderly adsorption process, probably due to strong interactions between the adsorbate and adsorbent. This comparative analysis underlines that the adsorption mechanism varies by material: SD and ST are more temperature sensitive due to their endothermic profiles, while CFs and SSs operate more efficiently at lower temperatures due to their exothermic nature, with SSs standing out for their high spontaneity and stability of adsorption.

### 2.8. Adsorption Mechanisms

**Figure 8:**
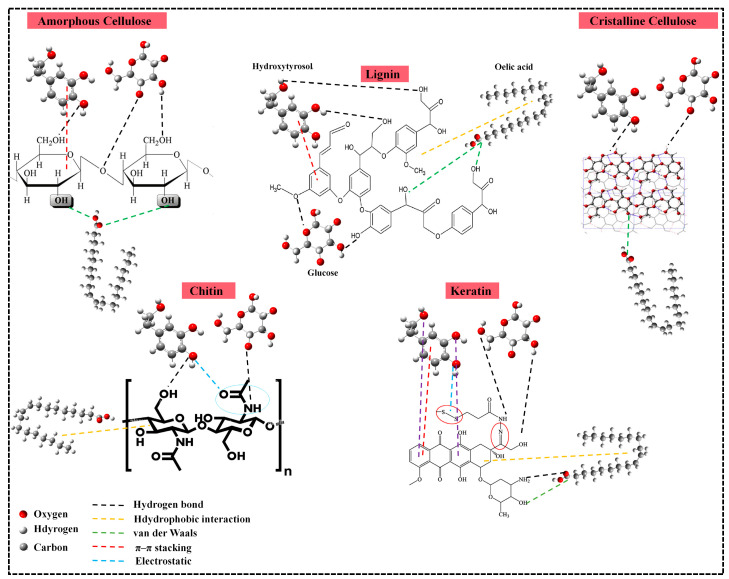
Adsorption mechanism of OMW organic matter into amorphous and crystalline cellulose, lignin, chitin, and keratin.

To elucidate the adsorption mechanisms involved in the removal of organic components from OMW, hydroxytyrosol, glucose, and oleic acid were selected as representative compounds based on their abundance, structural diversity, and polarity, encompassing both polar and non-polar characteristics, to assess their adsorption potential across various surface types [3,86]. SD and ST were modeled using lignin and cellulose (amorphous and crystalline), SSs were represented by chitin, and CFs were represented by keratin [64,73,87]. The experimental findings revealed that SD and ST exhibited an optimal performance observed at acidic pH = 4 for COD removal. Based on the findings discussed in the previous sections regarding the influence of operational parameters, a general mechanistic interpretation can be proposed, as illustrated in Figure 8. On lignocellulosic surfaces, particularly cellulose and lignin, hydrogen bonding and van der Waals interactions are likely to occur with the polar functional groups (-OH, -COOH) present in organic molecules such as glucose and hydroxytyrosol. In addition, π–π stacking between the aromatic rings of lignin and the phenolic rings of hydroxytyrosol may contribute to physical adsorption. Oleic acid, which contains both a polar carboxylic group and a long nonpolar chain, can interact through van der Waals forces with hydroxyl groups on lignocellulosic surfaces and through hydrophobic interactions with lignin. The predominance of these weak, non-specific interactions, combined with the limited number of reactive sites, likely explain the modest adsorption efficiency of SD and ST observed in the experimental data. Likewise, SSs achieved their highest COD removal efficiency at neutral pH (pH = 7), aligning with the amphoteric behavior of chitin. Chitin contains hydroxyl and amine groups that remain partially protonated or deprotonated at neutral pH, allowing for both electrostatic interactions and hydrogen bonding with diverse organic molecules containing hydroxyl groups. The acetamido and hydroxyl groups in chitin can form stable interactions with carboxyl and hydroxyl groups in glucose and hydroxytyrosol, while hydrophobic interactions with oleic acid further stabilize the adsorption process. The combination of these interactions explains the high performance of SSs at neutral pH and supports the occurrence of both physisorption and chemisorption. Then, CFs exhibited optimal adsorption at alkaline pH = 10, which enhances the deprotonation of keratin surface functional groups such as thiol and amino groups, leading to strong electrostatic attractions with the acidic components of OMW. At high pH, keratin’s negatively charged sites can effectively attract cationic or polar species, enhancing COD removal. Moreover, hydrogen bonding, n-π *, and π-π interactions with aromatic moieties such as hydroxytyrosol also contribute to the overall adsorption process.

### 2.9. Desorption Analyis

The assessment of the economic sustainability of OMW treatment by adsorption requires the recovery and regeneration of saturated adsorbents by desorption. This procedure is essential to evaluate the shelf life of the adsorbents and their ability to be used several times. The desorption analysis in Figure S6 highlights notable differences in the release behavior of COD across different adsorbents and tested media, revealing key adsorption mechanisms. At neutral pH, desorption is moderate, ranging between 20 and 26% for COD removal for all adsorbents, indicating that weak physical forces like hydrogen bonding and van der Waals interactions play roles in COD adsorption. The acidic condition (pH = 2) yields the highest desorption efficiency of COD for ST, which exhibits the highest desorption of rate at 46%, followed by CFs (45%); this suggests that electrostatic repulsion and the protonation of functional groups weaken adsorbate–adsorbent interactions, facilitating desorption and chemical binding as the most likely adsorption mechanism, as reported by Papaoikonomou et al. [58]. Under basic conditions (pH = 12), desorption efficiency is significantly lower, reaching only 24% for CFs, conversely achieving 55% for ST, 42% for SD, and 27% for SSs, confirming that strong ion-exchange and complexation mechanisms dominate the adsorption process of organic matter mainly onto ST and SD, in disagreement with Elayadi et al. [77], who indicated that the optimal desorption solution is acidic medium. Therefore, the effect of organic solvents demonstrates that methanol exhibits a higher desorption efficiency of COD than ethanol for ST and SS. Methanol desorption percentages are 62% for CFs and 72% for organic matter regeneration, while ethanol desorption is slightly lower at 52% (ST) and 41% (SSs). The superior desorption efficiency of methanol suggests that its smaller molecular size and higher polarity enhance the disruption of adsorbate–adsorbent interactions more effectively than ethanol [88]. The strong desorption observed in organic media confirms the contribution of hydrophobic interactions and chemisorption, particularly for SSs and ST, which exhibit the highest desorption rates. Overall, these findings indicate that acidic and organic media are the most effective for desorption, reinforcing the role of chemisorption in adsorption mechanisms and supporting the experimental data found. The potential regeneration and reuse of these adsorbents was proved.

### 2.10. Process Scalability and Cost

The economic viability and large-scale operation of adsorption for the treatment of OMW depends on the performance, affordability, and accessibility of the adsorbents. The four materials tested, SD, ST, CFs and SSs, are all inexpensive by-products and abundant in various industries, making them attractive candidates for large-scale applications. Each adsorbent has a specific cost and unique chemical and structural properties that influence its adsorption efficiency, regeneration potential, and overall ability to permanently treat OMW. They can be used in packed bed columns or batch reactors for industrialization. For the cost analysis, the cost per m^3^ of treated OMW was calculated using Equation (5):

(5) $$Cost\;per\;m^{3}=\frac{Adsorben\;tcost\;per\;Kg\times dosage\;per\;m^{3}}{COD\;removed\;per\;m^{3}}$$

The adsorbent costs (USD/kg) were based on current market prices of raw materials, and the dosages were those optimized during laboratory experiments according to the data given by the RSM-BBD approach. The adsorption capacities (mg COD/g of adsorbent) for SD, ST, CFs, and SSs (450, 575, 700, and 750 mg/g, respectively) were used to estimate COD removal per cubic meter of OMW. These calculations represent lab-scale estimations extrapolated for one cubic meter. The cost analysis of COD removal from OMW using different adsorbents highlights distinct differences in economic feasibility (Table S14). Firstly, CFs prove to be the most cost-effective option, requiring only 23.30 USD/m^3^ for COD removal, making it a highly economical choice. Then, SD, though slightly more expensive (59.4 USD/m^3^ for COD removal), still presents a reasonable balance between cost and efficiency. In contrast, ST exhibits the highest treatment costs (118.33 USD/m^3^ for COD removal), suggesting that despite its adsorption capacity, its high price may limit large-scale application. Hence, SSs while effective in pollutant removal, incur 87.48 USD/m^3^ for COD removal, making it a costlier alternative. Consequently, the adsorbent mixture (SD = 10%, ST = 28.9%, SSs = 38.3%, and CFs = 22.6%) emerges as a practical solution by optimizing costs and performance, with 45.47 USD/ m^3^ for COD removal toward the high cost of 87.48 USD/m^3^ obtained for individual SSs use as an efficient adsorbent for COD uptake. This suggests that combining different adsorbents enhance cost efficiency while maintaining effective organic matter removal, offering a viable approach for large-scale OMW treatment. Overall, the scalability of OMW treatment using these adsorbents depends on the appropriate compromise between adsorption efficiency, operating costs, and regeneration potential. Although these calculations are based on laboratory-scale data, the materials can be readily used in packed bed columns or batch reactors, making the process easily scalable. Future studies will focus on pilot-scale trials and complete cost modeling, including regeneration and operational expenses, and life-cycle assessments to better evaluate industrial applicability.

## 3. Materials and Methods

### 3.1. Olive Mill Wastewater (OMW) Sample Collection and Characterization

Olive mill wastewater (OMW) was collected from the three-phase continuous extraction during 2023–2024 in Beni-Mellal region in Morocco. The sample was stored in sterile, airtight containers and maintained at 4 °C to prevent microbial degradation and the oxidation reaction of polyphenols. A detailed physicochemical analysis was conducted to examine the organic and inorganic components of the samples. This involved measuring parameters such as pH, electrical conductivity (EC), total dissolved solids (TDSs), total solids (TSs), and fixed solids (FSs). Additionally, key metrics like total and dissolved chemical oxygen demand (TCOD and DCOD), polyphenol content, total Kjeldahl nitrogen (TKN), ammonium (NH_4_^+^), nitrate (NO_3_^−^), nitrite (NO_2_^−^), orthophosphate (PO_4_^3−^), chloride (Cl^−^), potassium (K^+^), sodium (Na^+^), sulfate (SO_4_^2−^), magnesium (Mg^2+^), and calcium (Ca^2+^) were evaluated. The pH was measured using a JENWAY 3505 pH meter, taffordshire, United Kingdom. while EC and TDSs were analyzed with a Mettler Toledo FiveEasy Benchtop F30 conductivity meter, manufactured by Mettler-Toledo GmbH, Greifensee, Switzerland. For determining TSs, FSs, and TDSs, the gravimetric method was applied [89]. The COD was measured using the potassium dichromate method, and the biological COD (BCOD_5_) was estimated using a respirometric method based on oxygen consumption by microorganisms over a 5-day incubation period at 20 ± 1 °C, as per the APHA standard [90]. Polyphenols were quantified using the Folin–Ciocalteu method [91]. TKN was calculated using the Kjeldahl method, and then the levels of ammonium, nitrate, nitrite, sulfate, and orthophosphate were assessed using AFNOR guidelines [92]. The concentrations of chloride, potassium, and sodium were determined through flame photometry (JENWAY), and calcium and magnesium levels were analyzed with the Rodier method by a complexation reaction [89]. This extensive analysis provides essential data on the OMW sample composition, which is crucial for evaluating the amounts of the organic and inorganic fractions.

### 3.2. Collection and Preparation of Raw Adsorbents

The raw adsorbents were prepared as follows:

Sawdust (SD) and straw (ST) were collected from local agricultural waste, washed with deionized water, dried at 105 °C for 24 h, ground, and sieved to obtain a uniform particle size (<630 µm).

Shrimp shells (SSs) were collected from seafood processing waste, washed with tap and deionized water, dried at 70 °C for 24 h, and ground.

Chicken feathers (CFs) were collected from poultry farms, washed with detergent to remove residual fat, dried at 50 °C, and ground into fine particles.

### 3.3. Adsorbent Characterization

The proximate analysis and component analysis of the biomass were determined by the method proposed in a previous study [93]. Each adsorbent was characterized to evaluate the structural and chemical modifications before and after adsorption. To evaluate the active site charges for all adsorbent and their difference in the adsorption of organic load from OMW, the point of zero charge (pHpzc) was determined by the solid addition method [94]. Boehm titration was also conducted to quantify the number of oxygenated groups for each material [95]. The nitrogen (N2) adsorption–desorption isotherms and pore size distribution were analyzed using an automatic surface area and pore analyzer (NOVA-4000, Quantachrome Instruments, Boynton Beach, FL, USA) at 77 K. The specific surface area (SSA) was calculated based on the Brunauer–Emmett–Teller (BET) equation. The crystalline structure of the adsorbents was analyzed using X-ray diffraction with a Bruker D4 Endeavor Diffractometer, employing CuKα radiation (λ = 15.40598 nm). The analysis was conducted at room temperature with a step angle of 0.02°, covering a 2θ range from 5° to 90°. To examine the functional groups of the adsorbents, Fourier-transform infrared (FTIR) spectroscopy was performed using a Shimadzu corp. 00677 spectrophotometer, with measurements taken within the wavenumber range of 4000 to 400 cm^−1^. Scanning electron microscopy (SEM) and energy dispersive X-ray spectroscopy (EDS) were used to investigate the surface morphology and composition of the adsorbents before and after adsorption. These analyses were carried out with an SH-5500P SEM operating at 20 kV. A gold coating was applied to the samples to improve imaging prior to SEM analysis.

### 3.4. Adsorption Experiments

#### 3.4.1. Batch Adsorption

A series of batch adsorption experiments were carried out to assess the ability of SD, ST, SSs, and CFs to remove COD from OMW. The selected ranges of operational parameters were based on preliminary trials and literature data to cover the conditions most relevant for OMW treatment: pH (4–10) to span acidic to alkaline conditions, adsorbent dosage (2–6 g) to evaluate low and high solid-to-liquid ratios, temperature (25–60 °C) to represent typical ambient and moderately elevated treatment conditions, contact time (1–6 h) to capture both rapid and slower adsorption kinetics, and stirring speed (100–250 rpm) to assess the influence of external mass transfer. These ranges ensured sufficient variability for the Box–Behnken Design (BBD) to evaluate the main and interaction effects on COD removal. For each experiment, 100 mL of OMW was placed in Pyrex flasks and incubated under controlled conditions, where temperature, contact duration, and shaking speed were precisely adjusted in an orbital shaker (Stuart SI500, Bibby Scientific, Staffordshire, UK). The initial pH of the solution was modified using 2.5 mol/L HCl or 10 mol/L NaOH to avoid significant changes in the treated OMW volume. After the adsorption process, the solution was filtered, and COD concentrations were analyzed to determine the adsorption performance. Then the removal efficiency (COD %) of these compounds and adsorption capacity (q_e_) were calculated using Equations (5) and (6):

(6) $$\%\;\mathrm{Rem}\;=\frac{\left(C_{o}-C_{e}\right)}{C_{o}}\times 100$$

(7) $$q_{e}=\frac{\left(C_{o}-C_{e}\right)V}{m}$$

q_e_ is the adsorption capacity (mg/g), C_o_ is the initial COD concentration of OMW (mg/L), C_e_ is the equilibrium COD concentration of OMW (mg/L), m is the adsorbent mass, and V is the OMW volume (L).

#### 3.4.2. Optimization and Modeling

a. Response Surface Methodology (RSM)—Box–Behnken Design (BBD).

The optimization of adsorption conditions was conducted using the Box–Behnken Design (BBD) within the Response Surface Methodology (RSM) framework. This approach allows for the systematic investigation of both individual and interactive effects of key process parameters on the removal of COD. The independent variables considered in this study were pH (A), adsorbent dose (B), temperature (C), contact time (D), and stirring speed (E), with their respective ranges provided in Table S1. The quadratic model was utilized to describe the adsorption behavior, enabling the prediction of the optimal conditions for maximizing pollutant removal. RSM was chosen due to its ability to efficiently model nonlinear relationships and minimize the number of experimental trials compared to traditional full-factorial methods. By analyzing iso-response curves and response surfaces, this approach provides valuable insights into the combined effects of multiple factors, ensuring a more precise optimization of adsorption performance. The developed mathematical model not only enhances our understanding of adsorption mechanisms but also helps determine the optimal operating conditions for practical applications. Additionally, the BBD experimental matrix, which consists of 46 experiments for each bio-adsorbent, as detailed in Table S2, ensures a thorough assessment of adsorption performance.

b. Mixture Design for the Synergistic Study.

To explore the synergistic effects of various bio-adsorbent combinations, a mixture experimental design (ME) was utilized. This method aimed to determine the optimal blend of SD, ST, SSs, and CFs for enhanced COD removal from OMW. The total sum of components in each experimental run equaled 100%. Therefore, the adsorbent proportions in the mixture were varied within a range of 0.1 to 0.7, allowing for diverse combinations to be tested. These intervals were strategically chosen to explore a wide array of potential adsorbent ratios, ensuring the identification of any synergistic effects that might enhance adsorption efficiency. The experimental matrix, as shown in Table S3, consisted of 15 experimental runs, each corresponding to a unique combination of the four adsorbents. For consistency, each material was dried, ground, and sieved to a uniform particle size of 100–200 µm. The components were then weighed according to the specified ratios and thoroughly mixed. Batch adsorption tests were then conducted by adding the prepared adsorbent mixtures into OMW samples, which contained known concentrations of COD. The experimental conditions, pH, temperature, adsorbent dose, contact time, and stirring speed, were based on a previous optimization realized by the prior Box–Behnken Design (BBD) used for individual adsorbents. The analysis of adsorption performance across 15 experimental runs was carried out under fixed operational conditions (pH = 7, adsorbent dose = 2 g, contact time = 2 h, temperature = 30 °C, and agitation speed = 200 rpm). This mixture design allowed for a comprehensive assessment of the adsorption efficiencies of various biomass combinations. The synergistic effects were determined by comparing the removal efficiencies of the mixtures to those of the individual adsorbents.

#### 3.4.3. Adsorption Isotherms, Kinetics, and Thermodynamics

In this study, the adsorption of COD from OMW was examined using several isothermal models, including Langmuir, Freundlich, Temkin, and Redlich–Peterson (R-P). These models were used to assess the adsorption capacity, surface heterogeneity, and the nature of the interactions between pollutants and the adsorbent [96]. In order to elucidate the adsorption mechanism and evaluate the COD removal rate, kinetic models such as pseudo-first order (PFO), pseudo-second order (PSO), Elovich, and the intra-particle diffusion model were applied [97,98]. These kinetic analyses enabled us to gain a better understanding of the rate control steps and adsorption dynamics. In parallel, the thermodynamic parameters Gibbs free energy (ΔG°), enthalpy (ΔH°), and entropy (ΔS°) were determined to explore the spontaneity, feasibility, and thermal nature of the adsorption process [99]. Calculations were based on the Van’t Hoff equation and associated thermodynamic parameters were performed to evaluate the temperature dependence of the adsorption process. Therefore, the isotherms, kinetics, and thermodynamic models are detailed in Supplementary Material Text S1.

### 3.5. Desorption

After determining the optimal adsorption conditions, the used adsorbents underwent desorption experiments to assess the release of organic matter under controlled laboratory conditions. The desorption process began with washing the adsorbents with water to remove any unadsorbed or loosely bound residues, followed by drying at 40 °C for 24 h. To examine the influence of pH on desorption efficiency, 1 g of each adsorbent was agitated in 100 mL of water adjusted to pH 2, 7, or 12 using 50% (*v*/*v*) acetic acid and NaOH. Additionally, 50% ethanol and methanol were used to evaluate the potential of polar organic solvents to enhance organic compound extraction. The desorption process was conducted at 100 rpm for 24 h, and the resulting filtrates were analyzed for the COD content to identify the most effective desorption conditions for recovering valuable organic compounds from each adsorbent.

### 3.6. Statistical and Data Analyses

All experiments were conducted in triplicate to ensure the accuracy and reliability of the results. A range of statistical parameters were used to evaluate model performance and validate the findings displayed in Supplementary Material Text S2.

## 4. Conclusions

Biopolymers are increasingly recognized for their environmental compatibility, low cost, and surface functionalities that make them attractive for OMW treatment. In this study, four natural biopolymer-rich materials, SD, ST, CFs, and SSs, were evaluated for their capacity to adsorb COD from OMW. The use of RSM with a Box–Behnken Design (BBD) proved to be an effective statistical tool for optimizing the operational parameters and understanding the combined effects of the factors influencing COD removal. The maximum COD removal efficiencies reached 69.81% for SD, 82% for ST, 86.27% for CFs, and 85.71% for SSs. These findings highlight SSs as promising low-cost adsorbents for effective COD reduction from OMW based on the higher adsorbent capacity of 750 mg/g. ST and SD showed adsorption primarily governed by physisorption mechanisms, while SSs and CFs exhibited a combination of physisorption and chemisorption, attributed to their protein and mineral contents. To enhance performance, a mixture design approach was applied to evaluate the synergistic effect of combining different biopolymers. The optimal formulation of SD (10%), ST (28.9%), SSs (38.3%), and CFs (22.6%) achieved a removal efficiency of 82% for COD. This combination allowed the exploitation of the structural and chemical diversity of the adsorbents to maximize interactions with the pollutants. A mechanistic interpretation was subsequently deployed to illustrate the interactions between bio-adsorbents and both the polar and non-polar organic compounds present in OMW. For polar molecules, the adsorption mechanism is governed by hydrogen bonding, electrostatic interactions, π-π stacking, and n-π interactions. In contrast, the adsorption of non-polar compounds is mainly driven by hydrophobic effects and van der Waals forces. Desorption studies carried out under various conditions demonstrated good regeneration potential. Moreover, an economic analysis underscored the practical advantages of the optimized mixture. The cost of COD treatment was reduced from USD 87.48 to USD 45.47 per m^3^ of treated OMW. In conclusion, the synergy between lignocellulosic, keratin, and chitin-based adsorbents enhances COD removal. Future perspectives include scaling up the process to pilot and industrial applications, developing regeneration strategies that maintain adsorption performance over multiple cycles, and conducting a complete techno-economic and life-cycle assessment.

## Figures and Tables

**Table 1:** The physicochemical characteristics of the raw OMW.

Parameter	Concentration
pH	5.2 ± 0.5
Conductivity (mS/cm)	15.79 ± 0.06
Total dissolved solids (TDSs) (g/L)	7.51 ± 0.02
Total suspended solids (TSSs) (g/L)	3.25 ± 1.33
BOD_5_ (g/L)	28.50 + 1.63
Total solids (TSs) (g/L)	55.45 ± 0.03
Fixed solids (FSs) (g/L)	33.18 ± 0.03
Chloride—Cl^−^ (mg/L)	0.00215 ± 0.004
Potassium—K^+^ (mg/L)	0.00329 ± 0.003
Phosphate—PO_4_^3−^ (g/L)	2.33 ± 0.05
Ca^2+^ (mg/L)	0.055 ± 0.004
Mg^2+^ (mg/L)	0.056 ± 0.003
TKN (g/L)	1.15 ± 0.03
Nitrite—NO_2_^−^ (mg/L)	0.00083 ± 0.0005
Nitrate—NO_3_^−^ (mg/L)	0.01218 ± 0.004
Sulfate—SO_4_^2−^ (g/L)	2.72 ± 0.02
Total COD (g/L)	223 ± 03.65
Dissolved COD (g/L)	152 ± 0.05
Polyphenols (g/L)	3.11 ± 0.06

**Table 2:** Proximate analysis of ST, SD, SSs, and CFs.

Proximate Analysis (wt.%)	ST	SD	SSs	CFs
Moisture	3.24	4.5	6	3
Ash	2.96	0.5	3	4.5
Volatile matter	70.23	73.65	70.98	76.5
Fixed carbon	23.57	21.35	20.02	16
Component analysis (wt.%)				
Lignin	39.45	20.32	0	0
Cellulose	35.79	60.47	0	0
Hemicellulose	20.47	15.63	0	0
Extractives	4.29	3.58	0	0

**Table 3:** Acid and basic group concentrations of ST, SD, CFs, and SSs.

Sample	Carboxylic Acids (mmol/g)	Phenolic Acids (mmol/g)	Lactonic Acids (mmol/g)	Total Acid Groups (mmol/g)	Basic Groups (mmol/g)
ST	4.75	1.98	0.65	7.38	0.75
SD	3.25	1.12	0.45	5.42	1.25
CFs	2.37	0.95	0.23	3.55	2.12
SSs	3.12	1.35	1.14	5.61	3.17

**Table 4:** BET Surface area and BJH distribution pores.

Sample Type	SD	ST	CFs	SSs
BET Surface area (m^2^/g)	0.2810	5.3215	0.5648	1.6740
BJH Pore Volume (cm^3^/g)	0.0583	0.0813	0.0305	0.0455
BJH Pore Size (nm)	3.688	3.822	6.757	5.895

## Data Availability

The data will be made available on request. The datasets used and/or analyzed during the current study are available from the corresponding author upon reasonable request.

## Supplementary Materials

The following supporting information can be downloaded at: https://www.mdpi.com/article/10.3390/ijms26167738/s1.
