# Supplementary material for: Unveiling the Adsorptive Potential of Natural Biopolymers for Olive Mill Wastewater Treatment: A Synergistic Approach Using RSM-BBD, Mixture Design, Kinetics, and Mechanistic Analysis
Source: Int J Mol Sci. 2025 Aug 11;26(16):7738. doi: 10.3390/ijms26167738 (PMC12386832; doi:10.3390/ijms26167738)
Supplement: Supplementary file 1 [file ijms-26-07738-s001.zip › ijms-3790024-supplementary.pdf]

Supplementary Material

Unveiling the Adsorptive Potential of Natural Biopolymers for Olive Mill Wastewater Treatment: A Synergistic Approach Using RSM-BBD, Mixture Design, Kinetics, and Mechanistic Analysis

Sabah Elamraoui [1], Nouhaila Asdiou [1], Rachid El kaim Billah [1], Mounir El Achaby [2], Said Kounbach [3], Rachid Benhida [3,4] and Mounia Achak [1,3,*]

[1] Science Engineer Laboratory for Energy, National School of Applied Sciences, Chouaïb Doukkali University, El Jadida 24000, Morocco; elamraoui.s@ucd.ac.ma (S.E.); asdiou.n@ucd.ac.ma (N.A.); elkaimbillah.r@ucd.ac.ma (R.E.k.B.)
[2] Materials Science, Energy, and Nano-Engineering (MSN) Department, Mohammed VI Polytechnic University (UM6P), Lot 660—Hay Moulay Rachid, Benguerir 43150, Morocco; mounir.elachaby@um6p.ma
[3] Chemical & Biochemical Sciences Green Process Engineering, CBS, Mohammed VI Polytechnic University, Ben Guerir 43150, Morocco; said.kounbach@um6p.ma (S.K.); rachid.b@um6p.ma (R.B.)
[4] Institut de Chimie de Nice CRNS UMR7272, Université Côte d'Azur, 28 Avenue Valrose, 06108 Nice, France
* Correspondence: achak.m@ucd.ac.ma; Tel.: +212-661474231

Supplementary Material

List of Supporting Information

Text S1. Adsorption isotherm, kinetic, and thermodynamic models

Text S2. Statistical and data analysis

Table S1. Parameters interval for BBD experiment

Table S2. Box Behnken design matrix of five variables

Table S3. Mixture design matrix

Table S4. Adsorption efficiency of different adsorbents (ST, SD, CF, and SC) in removing COD.

Table S5. ANOVA test of different adsorbents (ST, SD, CF, and SC) in removing COD.

Table S6. Statistical metrics of COD quadratic model for ST, SD, CF and SS.

Table S7. Experiment results for COD removal by mixture design.

Table S8. Quadratic model ANOVA test for COD removal.

Table S9. Cubic model ANOVA test for COD removal.

Table S10. Model descriptor for quadratic and cubic model of COD removal.

Table S11. Adsorption isotherm parameters for COD removal.

Table S12. Kinetic parameters for COD by PSO, PSO, Elovich, and Intraparticule diffusion models.

Table S13. Thermodynamic parameters of adsorption onto SD, ST, CF, and SS.

Table S14. Cost analysis for COD removal.

Figure S1: BET N_2 adsorption-desorption isotherm of SD, ST, CF, and SS.

Figure S2. Distribution of pores (PDS) by BJH model for SD, ST, CF, and SS.

Fig.S3. Normal plot of residuals (a) and externally studentized residuals (b) of SD, ST, CF and SS.

Figure S4. optimization for COD removal by RSM for SD, ST, CF, and SS.

Supplementary Material

Figure S5. Optimization by desirability function for mixture design

Figure S6. Desorption % at neutral (a), acidic (b), basic (c), organic medium (d-ethanol-e-methanol) onto SD, ST, CF, and SS.

Supplementary Material

Text S1. Adsorption isotherm, kinetic, and thermodynamic models

In this study, the adsorption of COD from OMW was examined using several isothermal models, including Langmuir, Freundlich, Temkin and Redlich-Peterson (R-P). These models were used to assess adsorption capacity, surface heterogeneity and the nature of interactions between pollutants and adsorbent [1]. In order to elucidate the adsorption mechanism and evaluate the COD rate removal, kinetic models such as pseudo-first order (PFO), pseudo-second order (PSO), Elovich and the intra-particle diffusion model were applied [2,3]. These kinetic analyses enabled to gain a better understanding of the rate control steps and adsorption dynamics. In parallel, thermodynamic parameters: Gibbs free energy (ΔG°), enthalpy (ΔH°) and entropy (ΔS°) were determined to explore the spontaneity, feasibility and thermal nature of the adsorption process [4]. Calculations based on the Van't Hoff equation and associated. Therefore, the isotherm, Kinetic, and thermodynamic models is detailed as follow:

a. Isotherm model:

- **Langmuir model** assumes monolayer adsorption on a homogeneous surface with identical adsorption sites. It is expressed as:

$$Qe = \frac{Q_m K_L C_e}{1+K_L C_e} \qquad \text{Eq S1}$$

Q_e (mg/g) is the amount of adsorbate per unit mass of the adsorbent at equilibrium, Q_m (mg/g) represents the maximum adsorption capacity, K_L (L/mg) is the Langmuir constant related to the affinity of the binding sites, and C_e (mg/L) is the equilibrium concentration of the adsorbate in solution. The separation factor (R_L), a dimensionless parameter that indicates the favorability of adsorption, is given by:

$$R_L = \frac{1}{1+K_L C_e} \qquad \text{Eq S2}$$

Where C_0 is the initial concentration of the adsorbate (mg/L). The adsorption is considered irreversible if $R_L=0$, favorable if $0<R_L<1$, linear if $R_L=1$, and unfavorable if $R_L>1$

Supplementary Material

-**Freundlich model** describes adsorption on a heterogeneous surface with varying adsorption site energies. It is represented as:

$$Qe = K_F \times C_e^{1/n} \qquad \text{Eq S3}$$

Where: K_F (mg/g)*(L/mg) is the Freundlich constant related to adsorption capacity, n is the heterogeneity factor, indicating adsorption intensity (adsorption is favorable if $1<n<10$).

-**Temkin model** accounts for adsorbate-adsorbent interactions and assumes that adsorption heat decreases linearly as coverage increases. It is given by:

$$Q_e = \frac{\mathrm{RT}}{\mathrm{b}} \ln\,(A_T C_e) \qquad \text{Eq S4}$$

Where: B=RT/b represents the adsorption heat. A_T (L/mg) is the Temkin isotherm constant, b (J/mol) is the Temkin constant related to adsorption energy, R (8.314 J/mol·K) is the universal gas constant, and T (K) is the absolute temperature.

-**Redlich-Peterson model** combines elements of both Langmuir and Freundlich models and can be applied to both homogeneous and heterogeneous adsorption. It is expressed as:

$$Q_e = \frac{K_R C_e}{1 + a_R C_e^g} \qquad \text{Eq S5}$$

Where: K_R (L/g) is the Redlich-Peterson isotherm constant, a_R (L/mg) is the R-P isotherm parameter, and g ($0 < g < 1$) is an exponent reflecting deviation from ideal monolayer adsorption.

b. Kinetic models:

-**Pseudo-first order model** is applicable for physisorption processes that dominate the adsorption mechanism, where adsorption occurs through weak Van der Waals forces and the

reaction rate is controlled by the adsorbate diffusion to the adsorbent surface. It follows Lagergren equation:

$$q_t = q_e(1 - e^{-K_1 t}) \qquad \text{Eq S6}$$

Where: q_t(mg/g) is the amount of adsorbate adsorbed at time t, q_e(mg/g) is the equilibrium adsorption capacity, and K_1 (min^{-1}) is the Pseudo-First Order rate constant.

-Pseudo-second order model assumes that the adsorption rate is proportional to the square of the available adsorption sites, suggesting a chemisorption mechanism. The model is expressed as:

$$q_t = \frac{{q_e}^2 K_2\, t}{1 + q_e K_2\, t} \qquad \text{Eq S7}$$

K_2 (g/mg·min) is the Pseudo-Second Order rate constant.

-Elovich model is used for describing adsorption processes where the surface is heterogeneous and activation energy varies with coverage. It is often used for systems involving chemisorption with slow diffusion and surface reactions. It is expressed as:

$$q_t = \frac{1}{b} \ln{(abt + 1)} \qquad \text{Eq S8}$$

a (mg/g·min) is the initial adsorption rate, and b (g/mg) is related to the desorption energy.

-**Intraparticle diffusion model** examines whether the adsorption process is controlled by diffusion within the adsorbent pores rather than surface reaction. It is given by:

$$q_t = K_{\mathrm{id}}\, t^{1/2} + C \qquad \text{Eq S9}$$

k_{id} (mg/g·$min^{1/2}$) is the intraparticle diffusion rate constant, and C (mg/g) is a constant related to the boundary layer effect.

c. Thermodynamic parameters:

Supplementary Material

-Gibbs free energy change (ΔG) is a key thermodynamic parameter that indicates the spontaneity of the adsorption process. If ΔG is negative, the adsorption process is spontaneous. If it is positive, the process is non-spontaneous. The more negative ΔG, the more favorable the adsorption process. It is determined using the following equation:

$$\Delta G = \Delta H - T\Delta S \qquad \text{Eq S10}$$

ΔG (kJ/mol) is the Gibbs free energy change, ΔH (kJ/mol) is the enthalpy change, which indicates the heat released or absorbed during the adsorption process, T (K) is the temperature in Kelvin, and ΔS (J/mol·K) is the entropy change, which reflects the disorder or randomness associated with the adsorption process.

-Van't Hoff equation is used to study the temperature dependence of the equilibrium constant and estimate the enthalpy change (ΔH) for the adsorption process. It can be written as:

$$Ln\ K_d = \frac{-\Delta H}{R} \times \frac{1}{T} + \frac{\Delta S}{R} \qquad \text{Eq S11}$$

Enthalpy change (ΔH) indicates whether is exothermic or endothermic and the entropy change (ΔS) reflects the disorder or randomness associated with the adsorption process.

Text S2. Statistical and data analysis

Analysis of Variance (ANOVA) was employed to assess the statistical significance of the experimental results, with a significance threshold set at $p < 0.05$. The coefficient of determination (R^2) and adjusted R^2 values were calculated to determine how effectively the model explained the observed data. To further examine the model predictive accuracy, Root Mean Squared Error (RMSE) was computed, offering a measure of how well the model predictions aligned with the experimental observations. The lack-of-fit tests were performed to ensure that the model adequately captured the underlying data patterns. For model comparison, both the Akaike Information Criterion (AIC) and Bayesian Information Criterion (BIC) were

Supplementary Material

calculated. These indices help strike a balance between the goodness of fit and the model complexity, with lower values of AIC and BIC indicating a more reliable and efficient model. AIC primarily focuses on reducing prediction error, while BIC imposes a greater penalty for more complex models, especially when larger sample sizes are involved. Together, these statistical techniques provided a robust framework for model validation, ensuring the integrity and precision of the experimental results.

Table S1. Parameters interval for BBD experiment

**Parameter**	**A**	**B(g)**	**C(°C)**	**D(h)**	**E(rpm)**
**Interval**	4-10	2-6	25-60	1-6	100-250

Table S2. Box Behnken design matrix of five variables

**Essai**	**A**	**B**	**C**	**D**	**E**
**1**	4	2	42,5	3,5	175
**2**	10	2	42,5	3,5	175
**3**	4	6	42,5	3,5	175
**4**	10	6	42,5	3,5	175
**5**	7	4	25,0	1,0	175
**6**	7	4	60,0	1,0	175
**7**	7	4	25,0	6,0	175
**8**	7	4	60,0	6,0	175
**9**	7	2	42,5	3,5	100
**10**	7	6	42,5	3,5	100
**11**	7	2	42,5	3,5	250
**12**	7	6	42,5	3,5	250
**13**	4	4	25,0	3,5	175
**14**	10	4	25,0	3,5	175

Supplementary Material

**15**	4	4	60,0	3,5	175
**16**	10	4	60,0	3,5	175
**17**	7	4	42,5	1,0	100
**18**	7	4	42,5	6,0	100
**19**	7	4	42,5	1,0	250
**20**	7	4	42,5	6,0	250
**21**	7	2	25,0	3,5	175
**22**	7	6	25,0	3,5	175
**23**	7	2	60,0	3,5	175
**24**	7	6	60,0	3,5	175
**25**	4	4	42,5	1,0	175
**26**	10	4	42,5	1,0	175
**27**	4	4	42,5	6,0	175
**28**	10	4	42,5	6,0	175
**29**	7	4	25,0	3,5	100
**30**	7	4	60,0	3,5	100
**31**	7	4	25,0	3,5	250
**32**	7	4	60,0	3,5	250
**33**	4	4	42,5	3,5	100
**34**	10	4	42,5	3,5	100
**35**	4	4	42,5	3,5	250
**36**	10	4	42,5	3,5	250
**37**	7	2	42,5	1,0	175
**38**	7	6	42,5	1,0	175
**39**	7	2	42,5	6,0	175
**40**	7	6	42,5	6,0	175
**41**	7	4	42,5	3,5	175
**42**	7	4	42,5	3,5	175
**43**	7	4	42,5	3,5	175

**44**	7	4	42,5	3,5	175
**45**	7	4	42,5	3,5	175
**46**	7	4	42,5	3,5	175

Table S3. Mixture design matrix

Run	A:SD	B:ST	C: SS	D:CF
**1**	0.175	0.175	0.175	0.475
**2**	0.4	0.1	0.1	0.4
**3**	0.4	0.1	0.4	0.1
**4**	0.475	0.175	0.175	0.175
**5**	0.1	0.4	0.4	0.1
**6**	0.175	0.475	0.175	0.175
**7**	0.175	0.175	0.475	0.175
**8**	0.1	0.1	0.4	0.4
**9**	0.1	0.1	0.7	0.1
**10**	0.7	0.1	0.1	0.1
**11**	0.1	0.7	0.1	0.1
**12**	0.1	0.4	0.1	0.4
**13**	0.4	0.4	0.1	0.1
**14**	0.1	0.1	0.1	0.7
**15**	0.25	0.25	0.25	0.25

Supplementary Material

Table S4. Adsorption efficiency of different adsorbents (ST, SD, CF, and SC) in removing COD

**Essai**	**COD %**			
	**ST**	**SD**	**CF**	**SS**
**1**	26.51	29.40	16.85	20.61
**2**	72.99	35.82	71.75	46.26
**3**	31.36	62.99	21.16	13.65
**4**	70.46	13.70	77.24	43.38
**5**	61.68	30.83	39.1	52.91
**6**	22.3	24.41	45.28	70.63
**7**	64.72	27.26	48.22	69.41
**8**	24.43	33.68	34.11	56.45
**9**	9.25	36.53	30.58	65.75
**10**	35.11	27.98	33.12	40.36
**11**	54.39	27.98	37.43	63.67
**12**	45.86	32.26	20.77	77.71
**13**	21.25	52,97	16.17	20.12
**14**	88.54	26.55	72.54	68.15
**15**	9.86	62.27	11.28	31.65
**16**	45.11	17.98	84.99	38.15
**17**	22.09	32.97	53.44	22.15
**18**	30.68	27.98	30.77	85.36
**19**	63.58	36.53	40.18	52.67
**20**	27.01	36.53	45.87	49.47
**21**	47.95	27.26	43.51	78.48

Supplementary Material

**22**	51.29	33.68	46.73	58.45
**23**	29.63	28.69	32.28	60.23
**24**	27.40	27.98	14.4	63.48
**25**	15.25	69.40	28.81	10.52
**26**	71.15	25.82	75.57	46.01
**27**	12.35	67.98	24.3	16.17
**28**	72.62	25.82	74.7	28.01
**29**	34.56	27.98	45.67	53.11
**30**	11.85	37.96	22.93	55.12
**31**	55.31	24,41	46.66	61.32
**32**	16.17	32.97	27.83	79.32
**33**	18.83	62.27	20.97	10.62
**34**	71.42	23.70	84.89	47.36
**35**	13.27	61.54	36.46	11.63
**36**	75.39	30.12	84.89	58.32
**37**	59.15	31.54	36.27	53.52
**38**	29.49	37.25	45.28	65.78
**39**	43.74	27.98	40.58	60.91
**40**	43.36	36.53	23.52	51.32
**41**	38.83	36.53	35.67	69.26
**42**	43.24	35.82	27.64	68.47
**43**	55.85	26.55	31.75	50.48
**44**	58.98	25.12	38.22	68.31
**45**	57.32	23.70	22.05	58.45
**46**	67.73	25.84	20.77	75.98

Supplementary Material

Table S5. ANOVA test of different adsorbents (ST, SD, CF, and SC) in removing COD

Source	COD							
	ST		SD		CF		SS	
	F value	P value	F value	P value	F value	P value	F value	P value
Model	24.10	<0.0001	20.02	<0.0001	15.72	<0.0001	21.84	<0.0001
A-pH	295.08	<0.0001	312.67	<0.0001	233.10	<0.0001	85.13	<0.0001
B-Mass	11.43	0.0024	0.9272	0.3448	0.8389	0.3685	1.84	0.1870
C-Temperature	84.79	<0.0001	1.39	0.2500	8.39	0.0077	0.8916	0.3541
D-Time	1.98	0.1719	0.0783	0.7819	2.01	0.1684	0.0001	0.9915
E-Stirring speed	0.0289	0.8664	0.0796	0.7802	0.3605	0.5536	14.99	0.0007
AB	1.48	0.2346	8.32	0.0080	0.0064	0.9369	0.1026	0.7513
AC	14.20	0.0009	1.77	0.1951	1.38	0.2510	10.13	0.0039
AD	4.51	0.0438	0.0254	0.8747	0.0609	0.8072	3.29	0.0819
AE	0.8039	0.3785	0.1022	0.7519	1.10	0.3039	0.5816	0.4528
BC	6.48	0.0174	0.6395	0.4314	2.04	0.1651	3.18	0.0865
BD	0.0496	0.8256	0.1015	0.7527	3.12	0.0895	2.83	0.1051
BE	6.96	0.0141	2.07	0.1626	1.69	0.2051	9.10	0.0058
CD	0.2277	0.6374	2.07	0.1622	1.89	0.1813	5.40	0.0286
CE	0.1028	0.7512	0.0254	0.8747	0.0702	0.7932	6.03	0.0214
DE	0.1248	0.7268	0.3132	0.5807	3.69	0.0661	46.59	<0.0001
A^2	52.53	<0.0001	56.32	<0.0001	46.65	<0.0001	205.54	<0.0001
B^2	2.35	0.1378	0.0039	0.9505	0.0457	0.8325	1.03	0.3191
C^2	4.61	0.0417	0.7379	0.3985	1.27	0.2698	2.77	0.1087
D^2	1.85	0.1863	6.47	0.0175	8.65	0.0070	8.28	0.0081
E^2	6.23	0.0195	1.65	0.2105	4.64	0.0410	0.3986	0.5335
Lack of Fit	0.4446	0.9110	0.515	0.867	1.09	0.511	0.3905	0.9398

Supplementary Material

Table S6. Statistical metrics of COD quadratic model for ST, SD, CF and SS

Parameters	CV %	R^2	R^2_{Adj}	R^2_{Pred}	AIC_C	BIC
**ST**	14.40	0.950	0.911	0.848	347.41	347.32
**SD**	12.94	0.941	0.894	0.814	320.50	320.41
**CF**	18.22	0.926	0.867	0.740	366.85	366.76
**SS**	12.56	0.945	0.902	0.837	395.49	395.39

Table S7. Experiment results for COD removal by mixture design

Run	A:SD	B:ST	C:SS	D:CF	COD %
**1**	0.175	0.175	0.175	0.475	45.24
**2**	0.4	0.1	0.1	0.4	48.75
**3**	0.4	0.1	0.4	0.1	45.88
**4**	0.475	0.175	0.175	0.175	52.34
**5**	0.1	0.4	0.4	0.1	92.13
**6**	0.175	0.475	0.175	0.175	65.61
**7**	0.175	0.175	0.475	0.175	64.98
**8**	0.1	0.1	0.4	0.4	35.24
**9**	0.1	0.1	0.7	0.1	68.92
**10**	0.7	0.1	0.1	0.1	39.65
**11**	0.1	0.7	0.1	0.1	58.65
**12**	0.1	0.4	0.1	0.4	40.12

Supplementary Material

**13**	0.4	0.4	0.1	0.1	53.97
**14**	0.1	0.1	0.1	0.7	27.58
**15**	0.25	0.25	0.25	0.25	54.56

Table S8. Quadratic model ANOVA test for COD removal

Source	Sum of Squares	df	Mean Square	F-value	p-value
**Model**	3513.90	9	390.43	47.66	0.0003
**(1)Linear Mixture**	2288.22	3	762.74	93.11	< 0.0001
**AB**	29.32	1	29.32	3.58	0.1171
**AC**	36.34	1	36.34	4.44	0.0890
**AD**	206.60	1	206.60	25.22	0.0040
**BC**	655.70	1	655.70	80.04	0.0003
**BD**	1.38	1	1.38	0.1687	0.6983
**CD**	98.30	1	98.30	12.00	0.0180
**Residual**	40.96	5	8.19		
**Cor Total**	3554.86	14			

Table S9. Cubic model ANOVA test for COD removal

**Model**	3528.30	13	271.41	9.32	0.2516
**(1)Linear Mixture**	2290.15	3	763.38	26.21	0.1424
**AB**	1.09	1	1.09	0.0375	0.8782
**AC**	1.20	1	1.20	0.0410	0.8728
**AD**	0.0424	1	0.0424	0.0015	0.9757
**BC**	30.18	1	30.18	1.04	0.4943
**BD**	3.70	1	3.70	0.1271	0.7820
**CD**	12.62	1	12.62	0.4334	0.6294
**ABC**	2.76	1	2.76	0.0948	0.8098

Supplementary Material

**ABD**	1.85	1	1.85	0.0636	0.8428
**ACD**	1.98	1	1.98	0.0681	0.8375
**BCD**	0.2297	1	0.2297	0.0079	0.9436
**Residual**	29.13	1	29.13		
**Cor Total**	3557.43	14			

Table S10. Model descriptor for quadratic and cubic model of COD removal

Response	R^2		Adjusted R^2	Predicted R^2	AIC	BIC
**Quadratic**	0.988		0.966	0.8112	112.07	82.45
**Cubic**	0.991		0.8854	-0.236	442.52	87.73

Table S11. Adsorption isotherm parameters for COD removal

	Redlich-Peterson				Langmuir				Freundlich			Temkin		
	K_{Red}	a	n	R^2	Q_m	K_L	R_L	R^2	K_F	n_F	R^2	B	A_t	R^2
	COD													
**SD**	4.46	0.001	1	0.978	386	0.001	7.69	0.97	5.53	1.08	0.976	232.2	0.05	0.961
**ST**	578.2	1.98	1	0.970	292.14	1.98	0.003	0.970	202	1.89	0.94	72.2	15.17	0.973
**CF**	510.02	0.060	1	0.99	839	0.060	0.12	0.99	184.7	3.38	0.968	169	0.71	0.985
**SS**	520.97	0.06	1	0.99	821	0.064	0.12	0.99	192.5	3.52	0.95	163.01	0.80	0.97

Supplementary Material

Table S12. Kinetic parameters for COD by PSO, PSO, Elovich, and Intraparticule diffusion models.

	PFO			PSO			Elovich			Intraparticule		
	**q_e**	**K_L**	**R^2**	**q_e**	**K_2**	**R^2**	**a**	**b**	**R^2**	**K_{diff}**	**C**	**R^2**
	**COD**											
**SD**	467	0.025	0.971	537.4	5.85	0.93	45.61	0.0092	0.970	24.35	109.56	0.69
**ST**	716.19	0.014	0.926	969.2	9.06	0.90	10.04	0.0039	0.87	42.15	6.95	0.83
**CF**	779	0.010	0.974	998	1.22E-6	0.970	14.20	0.03E-3	0.960	43.45	30.90	0.931
**SS**	751	0.011	0.983	946	0.064	0.978	15.83	0.004	0.968	41.62	45.50	0.934

Supplementary Material

Table S13. Thermodynamic parameters of adsorption onto SD, ST, CF, and SS

Adsorbent	T (°C)	ΔG (KJ/mol)	ΔH (KJ/mol)	ΔS (J/mol·K)
**SD**	25	16.232	70.843	-183.24
	40	13.488		
	60	-9.826		
**ST**	25	-13.078	30.030	144.66
	40	-15.248		
	60	-18.141		
**CF**	25	-19.674	-18.374	-5.483
	40	-16.683		
	60	-16.575		
**SS**	25	-24.929	-26.255	-7.805
	40	-23.81		
	60	-23.655		

Supplementary Material

Table S14. Cost analysis for COD and polyphenol

Adsorbent	COD	
	Cost (USD/kg)	Cost per m³ OMW Treated (USD)
**SD**	0.11	59.4
**ST**	0.28	118.33
**CF**	0.07	23.30
**SS**	0.27	87.48
**Mixture**	0.17	45.47

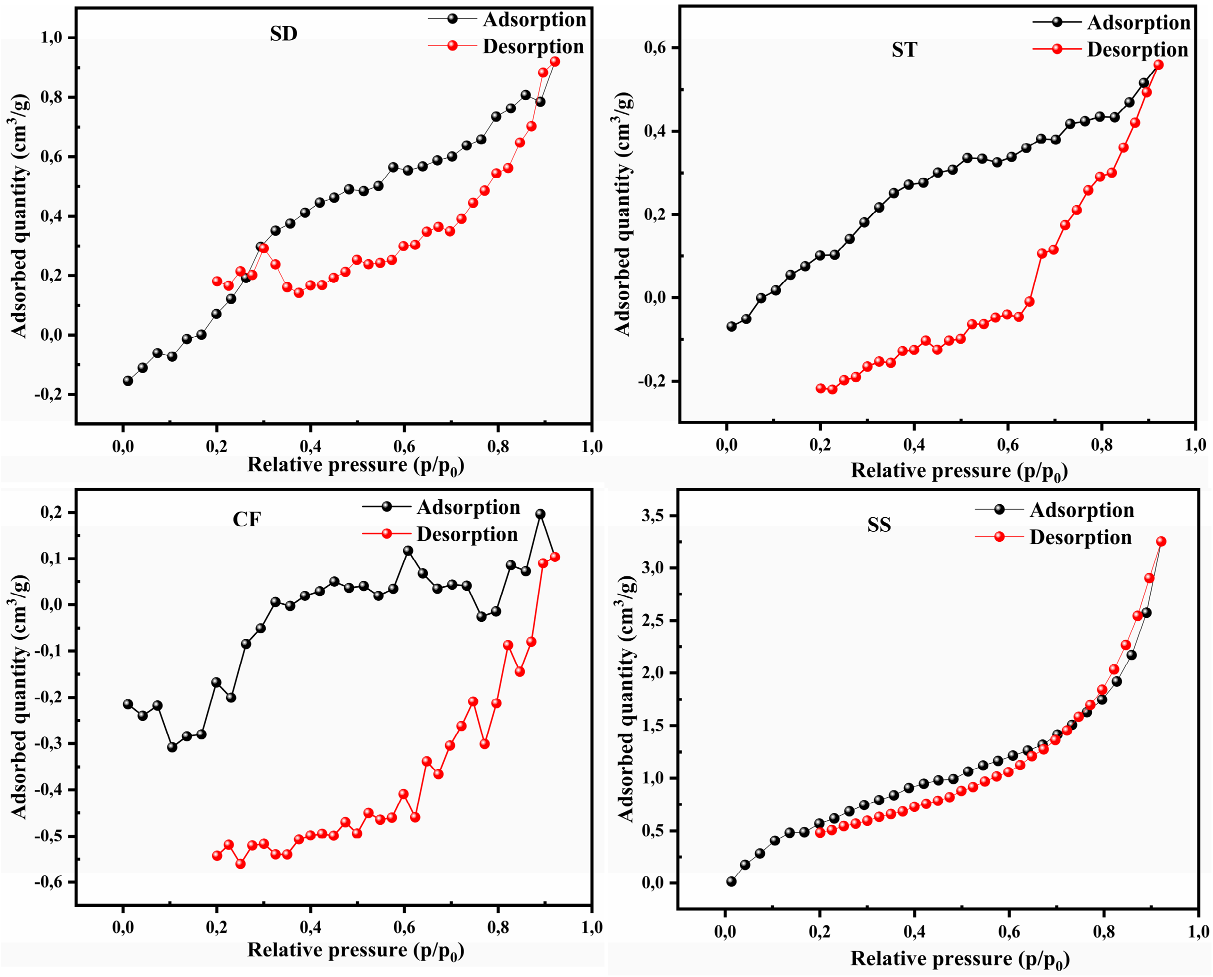

Figure S1. BET N_2 adsorption−desorption isotherm of SD, ST, CF, and SS.

Supplementary Material

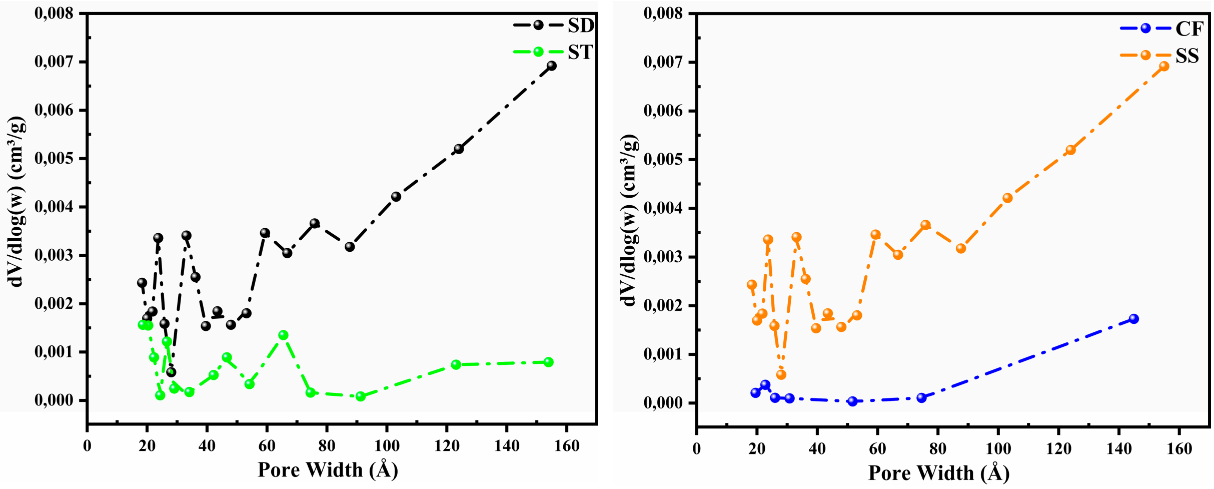

Figure S2. Distribution of pores (PDS) by BJH model for SD, ST, CF, and SC

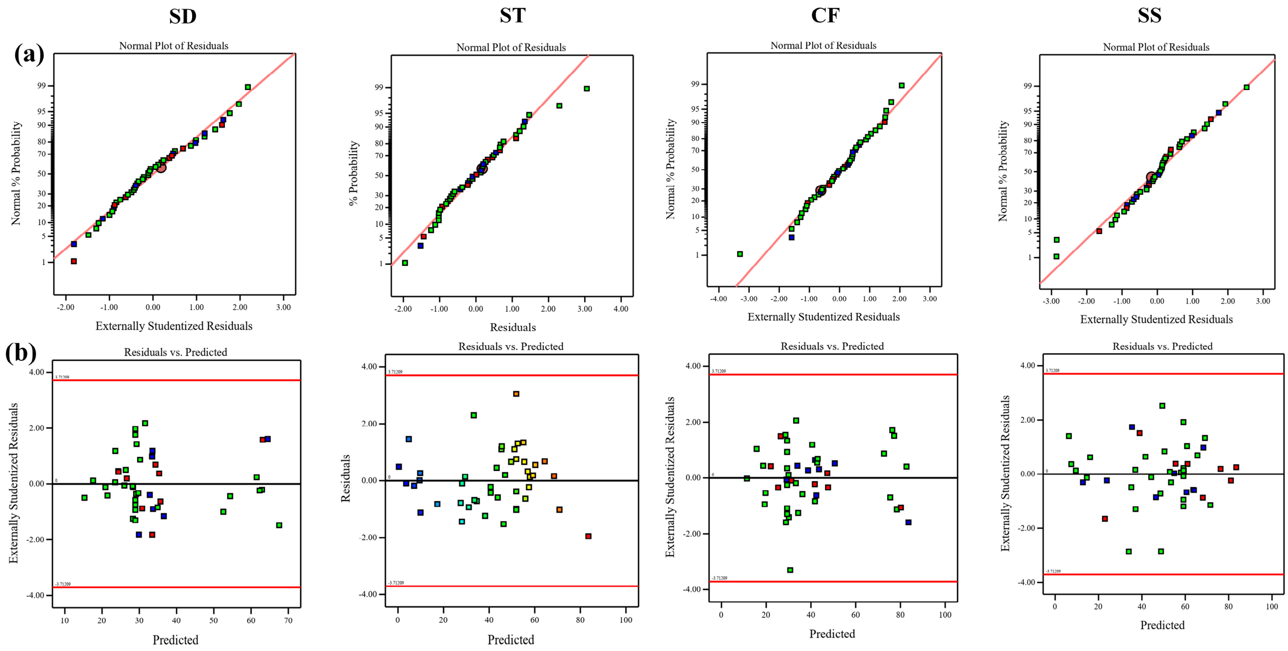

Figure S3. Normal plot of residuals (a) and externally studentized residuals (b) of SD, ST, CF and SS

Supplementary Material

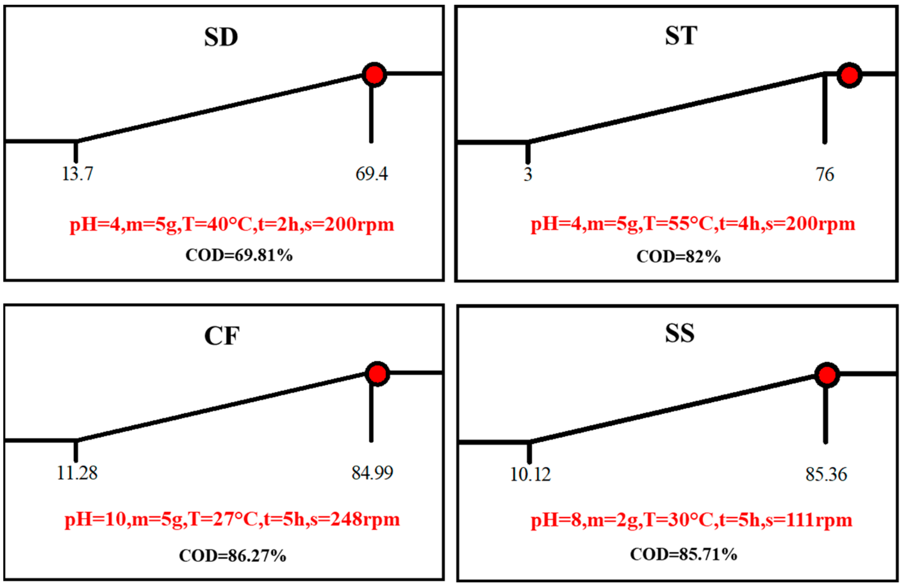

Figure S4. optimization for COD removal by RSM for SD, ST, CF, and SS.

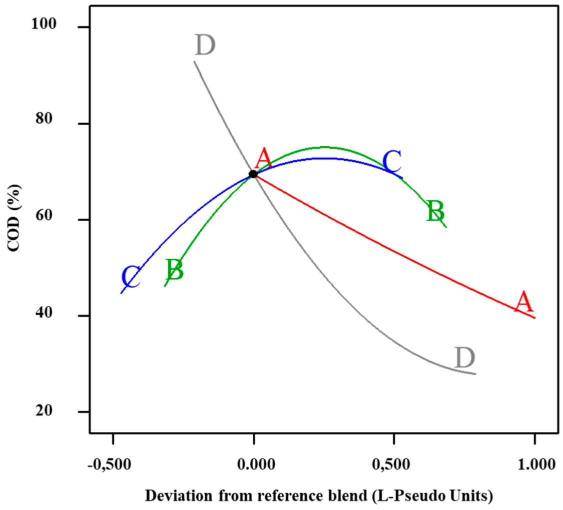

Figure S5. Optimization by desirability function for mixture design of A (SD), B (ST), C(SSs), and D (CFs),

Supplementary Material

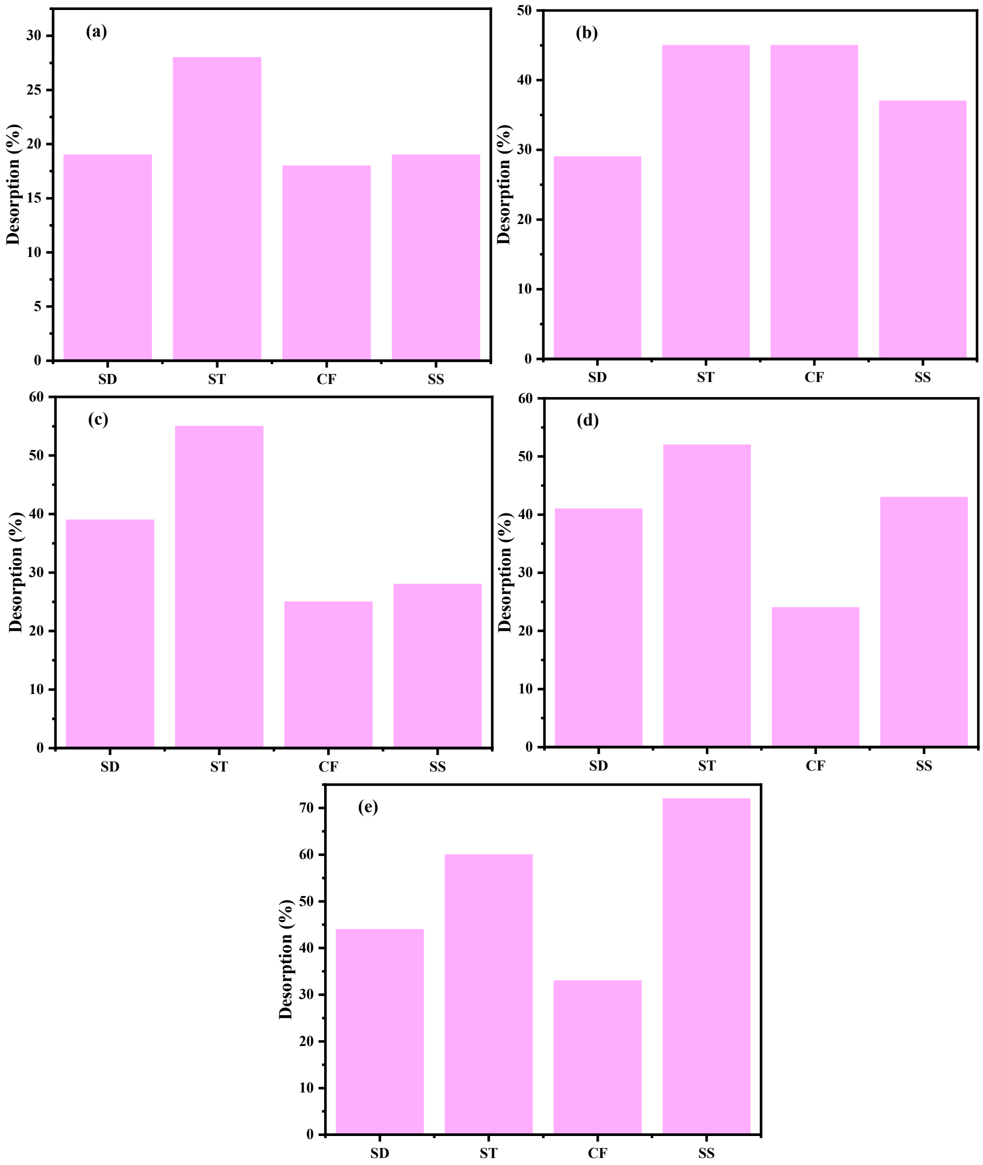

Figure S6. Desorption % at neutral (a), acidic (b), basic (c), organic medium (d-ethanol-e-methanol) onto SD, ST, CF, and SS.

Supplementary Material

References:

[1] Naboulsi A, Himri ME, Gharibi EK, Haddad ME. Study of adsorption mechanism of Malachite Green (MG) and Basic Yellow 28 (BY28) onto smectite rich natural clays (Ghassoul) using DFT/B3LYP and DOE/FFD. Surf Interfaces 2022;33:102227. https://doi.org/10.1016/j.surfin.2022.102227.

[2] Franco CA, Cortés FB, Nassar NN. Adsorptive removal of oil spill from oil-in-fresh water emulsions by hydrophobic alumina nanoparticles functionalized with petroleum vacuum residue. J Colloid Interface Sci 2014;425:168–77. https://doi.org/10.1016/j.jcis.2014.03.051.

[3] Zhang D, MacDonald L, Raj P, Karamalidis AK. Thiol-functionalized cellulose adsorbents for highly selective separation of palladium over platinum in acidic aqueous solutions. Chem Eng J 2024;494:152948. https://doi.org/10.1016/j.cej.2024.152948.

[4] Şahin S, Ciğeroğlu Z, Özdemir OK, Bilgin M, Elhussein E, Gülmez Ö. Recovery of hydroxytyrosol onto graphene oxide nanosheets: Equilibrium and kinetic models. J Mol Liq 2019;285:213–22. https://doi.org/10.1016/j.molliq.2019.04.097.
